# Modelling Hepatitis B virus related hospital discharges in Spain: ARIMAX based liver disease forecasting tool for hospital workload and mortality progression

**DOI:** 10.1371/journal.pone.0329751

**Published:** 2026-06-23

**Authors:** Lesly Acosta, Núria Soldevila, Núria Torner, Ana Avellón, Victoria Hernando, Eva Borràs, Ana Martínez, Carles Pericas, Cristina Rius, Pere Godoy, Angela Domínguez

**Affiliations:** 1 Universitat Politècnica de Catalunya· Barcelona Tech, Barcelona, Spain; 2 CIBER Epidemiología y Salud Pública (CIBERESP), Instituto de Salud Carlos III, Madrid, Spain; 3 Department of Medicine. University of Barcelona, Barcelona, Spain; 4 Hepatitis Unit, National Centre of Microbiology, Instituto de Salud Carlos III, Madrid, Spain; 5 Centro Nacional de Epidemiología. Instituto de Salud Carlos III, Madrid, Spain; 6 CIBER Enfermedades Infecciosas (CIBERINFEC), Instituto de Salud Carlos III, Madrid, Spain; 7 Agència de Salut Pública de Catalunya, Barcelona, Spain; 8 Agència de Salut Pública de Barcelona, Barcelona, Spain; 9 Institut de Recerca de l’Hospital de la Santa Creu i Sant Pau (IRB Sant Pau), Barcelona, Spain; 10 Universitat Pompeu Fabra, Barcelona, Spain; 11 Institut de Recerca Biomédica de Lleida (IRBLleida), Barcelona, Spain; Universidad Católica Sedes Sapientiae: Universidad Catolica Sedes Sapientiae, PERU

## Abstract

**Introduction:**

Chronic hepatitis B (CHB) virus infection leads to severe complications, cirrhosis and hepatocellular carcinoma (HCC). The main objective of this study was to develop an ARIMA-based model to forecast the progression of global hospital discharges, due to cirrhosis and HCC related to chronic hepatitis B.

**Methods:**

Retrospective observational study of monthly incidence of CHB related hospitalization discharges from 2005 to 2021 in Spain. Data were obtained through the Spanish Minimum dataset of hospital discharge registry (CMBDH) of the Health Ministry. Main diagnosis of CHB, liver cirrhosis and HCC encoded by International Medical codes (ICM–9 and ICM-10) were used. Descriptive and time series analysis was performed with forecasts made for 2022 using seasonal ARIMA and ARIMAX models. Data stationarity was achieved via a square root Box-Cox transformations and differencing. Model selection used was AIC, BIC, MAPE, and forecasting precision. Analysis was performed in R (version 4.5.0).

**Results:**

The total number of discharges related to hepatitis B was 6743, 58% were due to HCC and 42% to cirrhosis diagnosis. Median age was 59 years (range: 7 to > 100), being 83.4% men. The global chronic hepatitis B (CHB) related workload values range from 10 to 55 monthly discharges, while hepatitis B related to HCC and cirrhosis range from 4 to 34 and 1–29 discharges, respectively. The best fit and 2022 forecasts found for CHB and HCC time series was obtained with the approximate Gaussian $\sqrt{Y}$-ARIMAX (6,0,0) (0,1,1) _12 model. This model after treating outliers, removes seasonal patterns and captures the series’ autoregressive dynamics with an AR(6) and seasonal MA(1) noise with expression: $(\text{1}\;-\;\phi_{\text{1}}B\;-\;\phi_{\text{2}}B^{\text{2}}\;-\;...\;-\;\phi_{\text{6}}B^{\text{6}})(y_{t}\;-y_{t-\text{1}\text{2}})\;=\;\varepsilon_{t}\;+\;\Theta_{\text{1}}\;\varepsilon_{t-\text{1}\text{2}}$ with *ε* ~ N(0, σ²) and in the square root scale.

**Conclusion:**

Both ARIMA and ARIMAX models play critical roles in forecasting CHB-related HCC and cirrhosis, enabling better disease monitoring, healthcare resources, and intervention assessment. ARIMAX provided more accurate context-aware predictions, making it especially valuable for public health decision-making.

## Introduction

Viral hepatitis is one of the communicable diseases for which deaths are increasing, with an estimated 1.3 million deaths in 2022 worldwide. Hepatitis B virus (HBV) infection is responsible for 47% of these deaths. The regional distribution of viral hepatitis B varies, with 5% of the general population living with hepatitis B in the WHO African and Western Pacific Regions, while in the European and the Americas Region this prevalence is about 1% [1].

Hepatitis B virus is a DNA virus capable of integrating into the host genome inducing mutagenesis, leading to oncogene activation. HBV increases the risk of hepatocellular carcinoma (HCC) even in the absence of cirrhosis, although most patients with HBV- induced HCC have cirrhosis at presentation. HBV infection can be prevented by vaccination, and sequelae of chronic hepatitis B (CHB) infection—cirrhosis, liver failure, and HCC—can be prevented after the infection by antiviral therapy [2].

In 2016, the World Health Assembly approved the first global health sector strategy on viral hepatitis, being one of the main targets to reduce the burden of HBV infection and increased treatment in target population [3]. In 2019, WHO estimated there were 1.5 million new cases of CHB and 820,000 deaths related to HBV [3,4].

Burden of disease is not just the total number of new hepatitis infections in a population, but also the subsequent chronic infection and associated liver disease [5]. CHB infection is constitutes a significant public health threat globally, and a leading cause of cirrhosis, HCC and liver-related deaths [6].

Liver cancer remains a global health challenge and its incidence is growing worldwide. It is estimated that, by 2025, > 1 million individuals will be affected by liver cancer annually [2]. Hepatocellular carcinoma is the most common form of liver cancer and HBV infection is the most prominent risk factor for its development being the cause of approximately 50% of cases [7].

In Spain, [8]. twenty percent of viral hepatitis is caused by HBV infection and it represents an important hospital workload due to its main complications, liver cirrhosis and HCC, although a reduction in incidence and mortality have been observed during last decade [9–11].

A fundamental cornerstone in reducing the incidence of acute HBV infection has been the implementation of vaccination programs [12], first in adolescents and at risk population groups and since 2002, in newborns during the first year of life [11,13]. Vaccines used to prevent long-term clinical conditions, such as the hepatitis B vaccine, reduce the number of cancer cases many years after vaccination. Likewise, the appearance of new treatment alternatives should also be considered as variables that can modify the progression and evaluation results.

Mathematical modelling has become a valuable tool for the analysis of dynamics of infectious disease and for the support of control strategies development in recent years. Mainly, these modelling strategies deal on the analysis of transmission patterns, and methods to assess the effectiveness of control strategies for infectious diseases such as HIV, childhood infections, influenza, and vector borne infections [14]. Yet, to our knowledge there is little evidence of applied modelling for HCC and cirrhosis progression related to CHB infection.

There are several modelling methods used, such as Bayesian spatial model, machine-learning techniques, or regression-based models. In recent years, many methods were used to predict the incidence of infectious diseases, such as the Elman neural network model, or the dynamic linear ARIMA model applied in this work [15–17]. The ARIMA (Autoregressive Integrated Moving Average Model) statistical methodology is an important time series analysis tool for model estimation and prediction. Because it can capture the underlying trend, possible seasonality pattern and randomness of data, it is also used in the prediction of infectious diseases [18]. Yet, ARIMA extensions, known as ARIMAX models, allow for flexible modelling of different types of impacts; being a useful tool to evaluate the impact on prediction of exogeneous variables such as calendar effects, outliers presence or large-scale interventions [19,20].

The objective of this study is to propose a suitable ARIMAX type model to analyse and forecast the progression of liver disease complications (HCC and cirrhosis) related to chronic hepatitis B virus infection taking into account hospital discharge from the HB related admissions database from January 2005 to December 2021 in Spain.

## Materials and methods

### Study design and data collection

This retrospective study analysed hospital discharge data related to hepatitis B (HepB) from 2005 to 2021 in Spain. We considered the analysis of the global discharge data and also two sub-analyses corresponding only to HCC and to liver cirrhosis discharges. For each, the dataset was partitioned into two subsets: a training set (2005–2020) to build the ARIMA type model and a validation set (2021) to evaluate the models’ prediction performance; forecasts made for the year 2022. Data were obtained through the Spanish Minimum Dataset of Hospital Discharge registry (CMBDH) of Health Ministry on January 28^th^, 2025. The authors had no access to information that could identify individual participants during or after data collection. Main diagnosis for chronic hepatitis, liver cirrhosis and HCC encoded by the International Medical codes (ICM) –9 (for 2005−2016) and ICM-10 for 2017−2021 were used (Table 1), and the following secondary diagnoses of hepatitis B were used: 070.32 and 070.33 in ICD-9 and B18.0 and B18.2 in ICD-10. Variables included in the analysis were, age, sex, year/s of hospitalization, place of residence, date of admission and date of discharge. Patient consent was waived due to obtaining anonymized data from public health surveillance activities.

**Table 1:** Main diagnosis codes (ICM-9 or ICM-10) related to liver disease associated to chronic hepatitis B at discharge.

ICD-9
155.0 – Malignant neoplasm of liver, primary
571.40 – Chronic hepatitis, unspecified
571.41 – Chronic persistent hepatitis
571.49 – Other chronic hepatitis
571.5 – Cirrhosis of liver without mention of alcohol
571.8 – Other chronic non-alcoholic liver disease
571.9 – Unspecified chronic liver disease without mention of alcohol
**ICD-10**
C22.0 – Liver cell carcinoma (Hepatocellular carcinoma)
K72.1 – Chronic hepatic failure
K72.9 – Hepatic failure, unspecified
K73.9 – Chronic hepatitis, unspecified
K74.0 – Hepatic fibrosis
K74.2 – Hepatic fibrosis with hepatic sclerosis
K74.6 – Other and unspecified cirrhosis of liver
K75.3 – Granulomatous hepatitis, not elsewhere classified

## Statistical methodology

### Descriptive statistical analysis

Quantitative variables were summarized using the median (interquartile range, IQR). Categorical variables were described by frequency (percentage).

### Time series analysis

To explain and predict the primary outcome, “hospital discharges,” we first constructed a classic ARIMA (p,d,q)(P,D,Q)_S model, also known as Box Jenkins model [17]. In this model, p (P) represents the order of the autoregressive (seasonal autoregressive) component, indicating the number of lag observations used for prediction, d (D) is the order of regular (seasonal) differencing needed to achieve stationarity, q (Q) denotes the order of the moving average (seasonal moving average), representing the number of lagged forecast errors incorporated in the model. The argument S refers to the seasonal frequency, which is 12 for monthly data (i.e., 12 months per year).

Given that the ARIMA methodology requires stationary data, we previously applied a square-root transformation (Box–Cox with λ = 0.5). This served as a variance-stabilizing transformation (VST), which is the optimal approach for Poisson-distributed count data to stabilize the mean-variance relationship [21]. This transformation maps the discrete count process into an approximately continuous and homoscedastic scale, making it suitable for Gaussian ARIMA modelling [17]. Subsequently, we performed the necessary regular (order one) and seasonal (order S=12) differencing to ensure stationarity. After testing more than 5 models for each case, and based on the autocorrelation function (ACF) and partial autocorrelation function (PACF) patterns, we identified and fitted several plausible ARIMA (p,d,q)(P,D,Q)_S models to the hospital discharge data.

To enhance forecasting accuracy, the classic ARIMA approach was extended to a seasonal ARIMAX model, incorporating exogenous variables (X) to account in this case for outliers, particularly those related to the COVID-19 pandemic in 2020. Models were developed, based on the training subsets, for the global discharge time series data as well for the HCC and cirrhosis discharge data.

All models, both with and without outlier treatment, were validated using diagnostic tests, including the Box-Ljung test, Durbin-Watson test, normality tests, and homoscedasticity tests, to ensure their appropriateness for forecasting. To assist the identification and choice of the best invertible causal forecasting model validated with good fit and prediction metrics, we first analysed the data without including the pandemic period.

To select the most optimal model for the overall discharge data as well as for the HCC and cirrhosis components, we compared models using the Akaike Information Criterion (AIC), Bayesian Information Criterion (BIC), and the mean absolute percentage error (MAPE), which assess forecasting accuracy. Additionally, we introduced a proxy for forecasting precision, referred to as the “mean length” (ML), calculated as the mean difference between the upper (U) and lower (L) bounds of the confidence intervals across all time points in the validation set: ML = mean(U − L). Smaller values of all these measures indicate better model fit and forecasting performance. All statistical analyses were conducted using R (version 4.5.0).

### Ethical statement

No ethical approval was needed for this study because it was based on analysis of the data obtained from the MBDS (Minimum Basic Data Set) of the Ministry of Health of Spain. The data were completely anonymous and this study uses data with no identifiable information on the participants.

## Results

### Descriptive analysis

The total number of discharges related to hepatitis B from 2005 to 2021 was 6743, of these 3908 (58%) were due to HCC and 2835 (42%) to cirrhosis diagnosis. The median age of discharges was 59 years (range: 7–108), being 83.4% men and the three regions with higher occurrence were Andalusia with 1497 (22.2%) discharges, followed by Catalonia with 1469 (21.8%) and Madrid with 1174 (17.4%).

### Outcome measures

**Fig 1:**
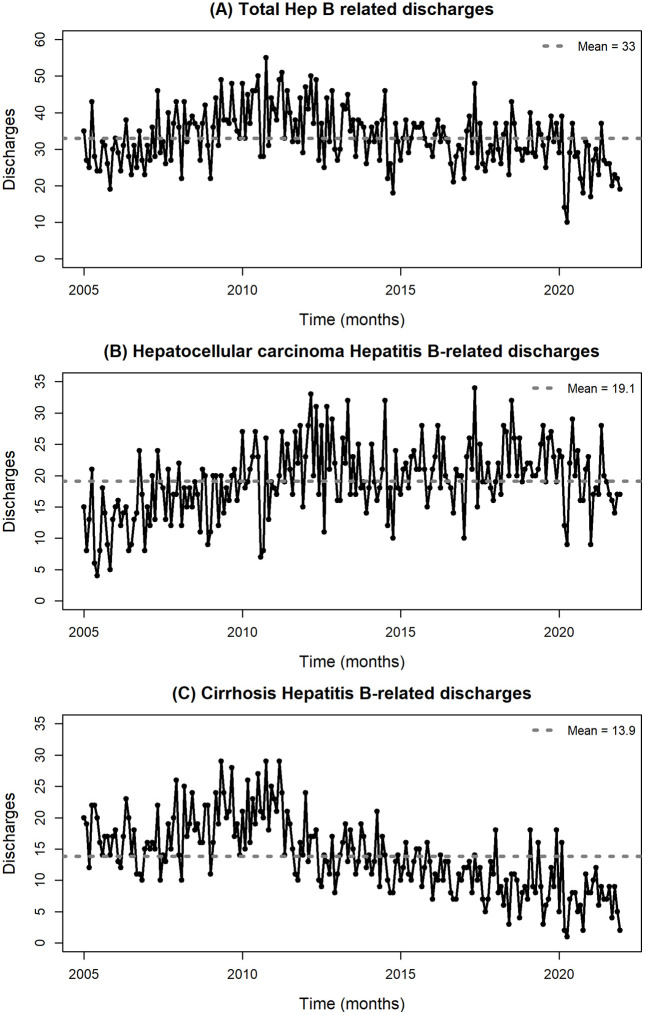
Evolution of chronic hepatitis B virus (HBV)-related hospital discharges: monthly time series data from 2005 to 2021 showing (A) total HBV discharges, (B) hepatocellular carcinoma discharges, and (C) cirrhosis discharges.

The primary outcome time series was the global CHB number of monthly hospital discharges. The analysis of secondary but relevant HCC and cirrhosis subsets data related to CHB were also explored. The global chronic hepatitis B related workload values ranged from 10 to 55 monthly discharges, while hepatitis B related to HCC and cirrhosis ranged from 4 to 34 and 1–29 discharges, respectively. Fig 1 shows the time series for total CHB related hospital discharges and those related to HCC and cirrhosis, from 2005 to 2021. Cirrhosis displayed a clearly diminishing trend while HCC did not.

### Time series analysis

**Fig 2:**
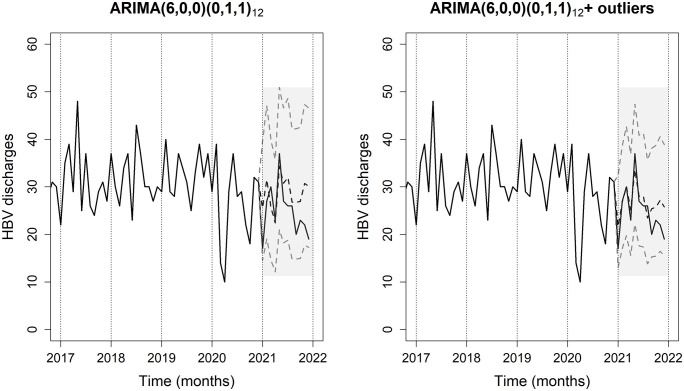
Out-of-sample comparison of ARIMA and ARIMAX models for HBV-related hospital discharges (train: 2005–2020; test: 2021). The shaded region shows ARIMAX’s narrower 95% confidence bands, indicating reduced prediction uncertainty.

**Fig 3:**
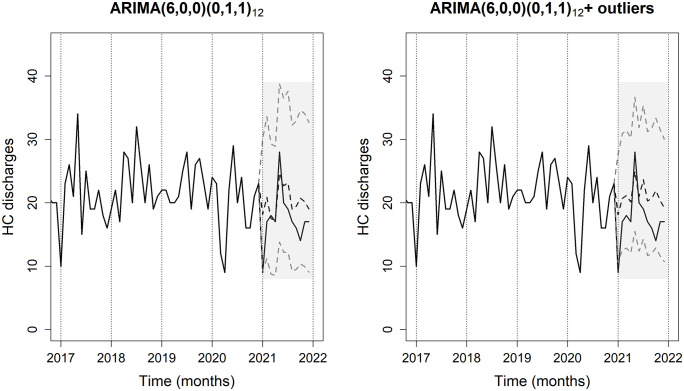
Out-of-sample comparison of ARIMA and ARIMAX models for HBV-related HCC discharges (train: 2005–2020; test: 2021). The shaded region shows ARIMAX’s narrower 95% confidence bands, indicating reduced prediction uncertainty.

The behaviour of hepatitis B virus time series analysis from 2005–2020 was studied and used to identify and fit different plausible ARIMA models. Then, known data from 2021 was used for model testing and finally forecast was performed for 2022. The best fit was obtained with an approximate Gaussian $\sqrt{Y}$ **-**ARIMA(6, 0, 0) (0, 1, 1)_12 model, which incorporates information of six previous months and one seasonal innovation term (Fig 2). While the data consists of counts, the volume of monthly cases (ranging from 10 to 55) was sufficient to support a continuous approximation, as the Poisson distribution P(λ) approaches normality when λ ≥ 10 [22,23]. Furthermore, the mean of the transformed series remained far from the zero boundary, rendering the probability of negative predictions statistically negligible. The parameters were estimated via Quasi-Maximum Likelihood Estimation (QMLE), which provides consistent estimates for ARMA-type models even if the true distribution deviates from Gaussianity, provided the conditional mean and variance are correctly specified [24].The assumption that the residuals (*ε*ₜ) behave as Gaussian white noise (zero mean, constant variance and no-autocorrelation structure) was validated through post-estimation diagnostic analysis of the residuals. The fitted model captures the series autoregressive dynamics with an AR(6) and a seasonal noise with seasonal MA(1) with expression: (1 − 𝜙_1_ 𝐵 − 𝜙_2_ 𝐵^2^ − ... − 𝜙_6_ 𝐵^6^) (𝐵_t_ − 𝐵_t-12_) = 𝜀_t_ + 𝛩₁ *ε*_t-12_ with *ε*  ~ N(0, σ^2^), being 𝐵ₜ the square root of non-zero counts. The same model holds in the stratified analysis when considering HCC as shown in Fig 3. When incorporating the treatment of outliers (ARIMAX) using the global data, five outlier values were detected with two occurring in the COVID-19 pandemic year; for HCC data eight outliers detected including two during the COVID-19 pandemic year. As shown in Figs 2 and 3 (right panels), more precise predictions are obtained with the ARIMAX model extension for both, the entire data set and the HCC data, respectively. Note the narrower confidence bands when treating outliers via the suitable fitted ARIMAX extension.

For cirrhosis, both best fit ARIMA and ARIMAX models of the form ARIMA (1,1,1) (0,1,1)_12 did not perform satisfactorily to explain or forecast cirrhosis discharges in 2021. It had a large relative mean absolute percentage prediction error of approximately 50%. It is confirmed that slightly more precise prediction intervals are obtained with the ARIMAX extension, but the bad forecasting ability remains with MAPE around 50% (bottom block, Table 2).

**Table 2:** Summary of adjustment and prediction ability metrics for three Hepatitis B related hospital discharge data sets.

Dataset	Model	Noise variance	AIC	BIC	MAPE	ML
**Global Hepatitis B**	ARIMA(6,0,0)(0,1,1)_12	0.388	392.49	418.55	0.234	27.59
	ARIMA(6,0,0)(0,1,1)_12 + outlier’s treatment	0.272	336.75	379.07	0.139	22.76
**HCC**	ARIMA(6,0,0)(0,1,1)_12	0.377	385.53	411.59	0.280	23.09
	ARIMA(6,0,0)(0,1,1)_12 + outlier’s treatment	0.256	330.35	385.68	0.240	19.41
**Cirrhosis**	ARIMA(1,1,1)(0,1,1)_12	**0.359**	**373.25**	**386.26**	**0.495**	**11.91**
	ARIMA(1,1,1)(0,1,1)_12 + outlier’s treatment	**0.339**	**377.42**	**419.70**	**0.488**	**11.61**

Summary performance metrics for adjustment and prediction ability are reported in Table 2; top block displays results for global data, middle block for subset HC data and bottom block for CI data. We observed that overall, the ARIMAX models had a much better performance. For the global and HCC data, all reported metrics improve (decrease) when considering the ARIMA modelling plus outliers’ treatment. The results for the global time series data can be observed in top block of Table 2 with goodness-of-fit metric given by the Akaike information criterion (AIC) dropping from 392.5 to 336.7 and relative MAPE prediction error decreasing from 23.4% to a good forecasting value of 13.9% [21]. The corresponding graphical comparative analysis of hepatitis B related HCC discharges according to performance of the ARIMA model versus de ARIMAX time series model during the time series 2005–2020 period to test forecasting capability as to 2021 was shown in Figs 2 and 3 respectively.

**Fig 4:**
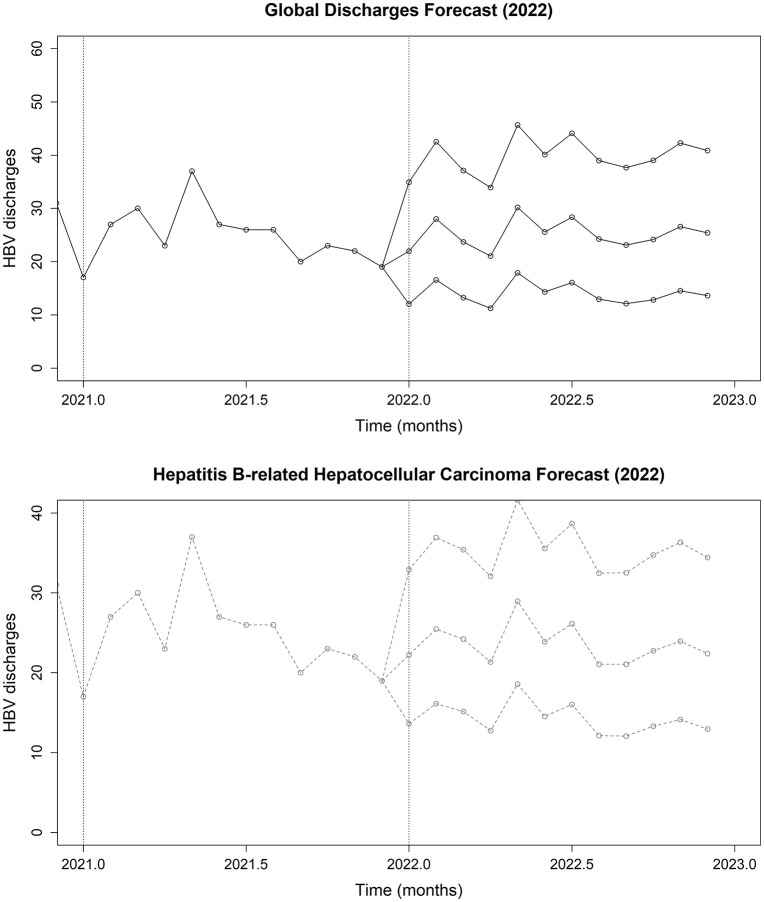
Forecasts of 2022 discharges for two series: global (top) and hepatitis B-related HCC (bottom). Comparative forecasting performance of ARIMA and ARIMAX (which includes outlier treatment) models.

Finally, the suitable found ARIMA and ARIMAX models for forecasting year 2022 discharge values for total hepatitis B data (left panel) and HCC data (right panel) are shown in Fig 4. In contrast to classic ARIMA, ARIMAX models yield best performance; particularly most precise discharge forecasts for year 2022. Furthermore, the suitability of the continuous approximation was confirmed by assessing the risk of boundary effects: it was verified that all in sample fits, out-of-sample predictions and forecasts were strictly non-negative (Figs 2-4). Given the mean (μ) and the estimated variance (σ^2) of our transformed series, the probability of the model generating a negative value was statistically negligible (the zero boundary lied far away from the mean). This indicates that the model respected the natural bounds of the data despite not imposing an explicit non-negativity constraint.

## Discussion

This study grants evidence as to the utility of ARIMA and ARIMAX modelling in forecasting hepatitis B-related HCC and cirrhosis as a predictor of hospital workload. Both models are widely used in medical and epidemiological forecasting. When applied to hepatitis B-relatedHCC and cirrhosis, these models can help predict disease trends, guide healthcare planning, and improve early intervention strategies. ARIMA models can analyse time-series data of HCC and cirrhosis cases over several years, capturing underlying trends, seasonality, and random fluctuations. These models can predict the future burden of hepatitis B-related complications, helping healthcare policymakers allocate resources effectively. The advantage of ARIMAX models, because they allow to incorporate exogenous variables (such as vaccination rates, antiviral therapy uptake, socioeconomic factors), is to improve mostly forecasting precision and possibly accuracy. For instance, including hepatitis B vaccination coverage could reveal how immunization impacts long-term HCC and cirrhosis trends. In our study, ARIMAX, by integrating relevant external factors, provided more precise and context-aware predictions, making it especially valuable for public health decision-making.

The incidence of hepatitis B is decreasing globally, but HBV still causes three-quarters of the 1.1 million annual deaths from hepatitis B and C, mainly due to HCC and cirrhosis. Twenty countries bear most of the global burden, with China, India, and Nigeria being the top three. Recent reports of the Coalition for Global Hepatitis Elimination [22] indicate that 75% of countries have met the 2025 WHO target for low HBsAg prevalence in young children, and two-thirds have universal newborn vaccination policies. However, only a few countries have met the diagnosis target, and none have reached the treatment target. While no country is on track to meet all HBV eradication targets by 2030, over 80 countries, including Spain, are on track to achieve elimination goals, particularly in regions with long-standing high vaccination coverage [22].

Several studies have observed a gradual decrease in cirrhosis cases, while the reduction in HCC incidence is less pronounced suggesting that although there is a slow reduction in cirrhosis cases, the decrease in HCC incidence is less evident, highlighting the need for continued efforts in prevention, early detection, and treatment strategies [15,23]. A study in Asian patients with chronic hepatitis B, tenofovir (TDF) therapy was significantly associated with a reduction in the 8-year cumulative incidence rate of HCC. However, the decline in HCC incidence is less pronounced compared to the reduction in cirrhosis cases [15,24–26]. While a study in Europe in the United States indicates that antiviral therapy has been shown to reduce the incidence of HCC among patients with chronic HBV or HCV, the overall reduction in HCC mortality rates is not as significant, suggesting that while cirrhosis cases may be declining, HCC remains a critical concern [27–29].

This study presents several limitations. First of all, the use as data source being the anonymous hospital discharge data does not allow for checking duplicates, encoding errors or other possible errors, yet being this a constant issue throughout the time series it should not affect forecasting capacity. While we employed a Gaussian ARIMAX framework supported by variance-stabilizing transformations, we acknowledge the potential utility of specialized discrete time series models (e.g., INGARCH or Poisson-based models). However, for the sample size and count levels observed in this study, the VST-ARIMAX approach offers a more parsimonious and numerically stable estimation, avoiding the convergence challenges often associated with non-linear MLE procedures in moderate-frequency [24,30]. Under the Quasi-Maximum Likelihood (QML) framework, these estimates remain consistent even if the underlying distribution is discrete, provided the conditional mean and variance are correctly specified [24]. Another limitation is the fact that no immunization in front of hepatitis B virus can be assessed and thus the protective effect of the vaccine cannot be graded, yet being HCC and cirrhosis a long-term consequence of HB infection, cohorts included ranging from 2005 to 2021 and considering incubation periods for these conditions to be at least 20 years, affected cases would be out of inclusion in generalized immunization programs.

## Conclusion

Hepatocellular carcinoma and cirrhosis are long-term consequences of HB infection. Both ARIMA and ARIMAX models play critical roles in forecasting HB-related HCC and cirrhosis, enabling better disease monitoring, healthcare resource allocation, and intervention assessment. However, ARIMAX provides more accurate and context-aware predictions, making it especially valuable for public health decision-making.
