# Correction: Modelling Hepatitis B virus related hospital discharges in Spain: ARIMAX based liver disease forecasting tool for hospital workload and mortality progression

**DOI:** 10.1371/journal.pone.0354253

**Published:** 2026-07-17

**Authors:** The PLOS One Staff

There are errors in the author affiliations. The correct affiliations are as follows

Lesly Acosta^**1**^, Núria Soldevila^**2,3**^, Núria Torner^**2**^, Ana Avellón^**2,4**^, Victoria Hernando^**5,6**^, Eva Borràs^**2,3,7**^, Ana Martínez^**2,7**^, Carles Pericas^**2,8,9**^, Cristina Rius^**2,8,9,10**^, Pere Godoy^**2,11**^, Angela Domínguez^**2,3**^

1 Universitat Politècnica de Catalunya· Barcelona Tech, Barcelona, Spain, 2 CIBER Epidemiología y Salud Pública (CIBERESP), Instituto de Salud Carlos III, Madrid, Spain, 3 Department of Medicine. University of Barcelona, Barcelona, Spain, 4 Hepatitis Unit, National Centre of Microbiology, Instituto de Salud Carlos III, Madrid, Spain, 5 Centro Nacional de Epidemiología. Instituto de Salud Carlos III, Madrid, Spain, 6 CIBER Enfermedades Infecciosas (CIBERINFEC), Instituto de Salud Carlos III, Madrid, Spain, 7 Agència de Salut Pública de Catalunya, Barcelona, Spain, 8 Agència de Salut Pública de Barcelona, Barcelona, Spain, 9 Institut de Recerca de l’Hospital de la Santa Creu i Sant Pau (IRB Sant Pau), Barcelona, Spain, 10 Universitat Pompeu Fabra, Barcelona, Spain, 11 Institut de Recerca Biomédica de Lleida (IRBLleida), Barcelona, Spain.

In the Time series analysis subsection of the Results, there is an error in the eighth sentence of the first paragraph. The correct sentence is: The fitted model captures the series autoregressive dynamics with an AR(6) and a seasonal noise with seasonal MA(1) with expression: (1 − *Φ*_1_ 𝐵 − *Φ*_2_ 𝐵² − ... − *Φ*_6_ 𝐵⁶) (*y_t_* − *y_t_* ₋ ₁₂) = *ε*_*t*_ + *Θ*_1_ ε_t_ ₋ ₁₂ with *ε* ~ N(0, σ²), being *y_t_* the square root of non-zero counts.

The publisher apologizes for the errors.
