# Supplementary material for: Modelling Hepatitis B virus related hospital discharges in Spain: ARIMAX based liver disease forecasting tool for hospital workload and mortality progression
Source: PLoS One. 2026 Jun 23;21(6):e0329751. doi: 10.1371/journal.pone.0329751 (PMC13289951; doi:10.1371/journal.pone.0329751)
Supplement: Attachment — Submitted filename: Response to Reviewers.docx [file pone.0329751.s002.docx]

**Manuscript title:** “Modelling Hepatitis B virus related hospital discharges in Spain: ARIMAX based liver disease forecasting tool for hospital workload and mortality progression”

**Response to Editor and Reviewers**

Dear Editor:

We greatly appreciate your interest in our work and the opportunity to improve the manuscript. We have revised the paper according to the reviewers’ suggestions, with all changes highlighted in red in the updated version. We have also prepared a detailed response letter in which each reviewer comment is reproduced, followed by our response in italic text. We also thank you for your feedback and help in this process.

**REVIEWER #1**

Dear Reviewer,

First of all, we would like to thank you for your valuable comments and suggestions, which have greatly helped us to improve the manuscript. We have incorporated all your recommendations into the new version of the document and highlighted them in red for easy identification. Below, we will respond to all of your comments. We will use an italic font for this purpose.

Additional notes/remarks:

- Below, we addressed all central points made by two reviewers and that you kindly summarized for us.
- As required, all plots were removed from within the manuscript. See attached .tiff files and corresponding captions (List of Figures and captionsLA.docx).
- We created a “Revised Manuscript with Track Changes.docx” document with red-marks to be uploaded separately. Also, we attach the unmark version in the “Manuscript.docx” File.

**Reviewer #1 comments**

**Here are my comments**

1) **Abstract:** Kindly report the confidence interval for the total number of discharges, percentage attributed to HCC, and cirrhosis to provide a better narrative of what is happening in the target population beyond the sample.

*The percentage attributed to HCC, and cirrhosis is provided in* ***line 53*** *of the abstract. To provide a better narrative of what is happening in the target population beyond the sample, we only aim to provide point predictions (and corresponding 95% CI to quantify uncertainty) via the classic ARIMA and ARIMAX extension method****.*** *However, although we do not make any inference on discharge data metrics, following your comment to provide a better general description of the three times series,* ***we added the phrase: “****The global chronic hepatitis B (CHB) related workload values range from 10 to 55 monthly discharges, while hepatitis B related to HCC and cirrhosis range from 4 to 34 and 1 to 29 discharges, respectively” (****lines 56-58****; see Fig. 1 also).*

2) **Abstract:** This statement is not result but methodology: “CHB time series analysis with data from 2005‐2020 was used to fit plausible ARIMA models, followed by testing with known 2021 data to obtain 2022 forecasts”

***You are right, thank you.*** *We removed such statement from the abstract and checked that this information/content was already incorporated in section “Materials and methods”.*

3) **Abstract:** The best fit was obtained with the model, except that cirrhosis should be revised. It sounds incomplete

***You are right, the sentence was incomplete.*** *We modified it to be complete and more clearly stated, by adding in lines x-x, the sentence: “The best fit and 2022 forecasts found for CHB and HCC time series was obtained with model ARIMAX (6,0,0) (0,1,1)_12.”* ***(lines 58-59).***

4) **Abstract:** The **results presented in the abstract section** are just descriptive summary measures, and nothing about the results from the actual modelling study. Kindly present the key results of the modelling study. What was the best predictive model based on the model performance measures? How did the observed data in 2021 compare with the predicted data from the model in 2021? What was the forecasted estimate of the outcome measure and corresponding confidence interval in 2022 Methods

*Now, apart from key results of the modelling and forecasting study provided before (following your 3^rd^-comment),* ***we added a sentence*** *regarding the functional form of the chosen model: “This model after treating outliers, removes the seasonal patterns through seasonal differencing and captures the series autoregressive dynamics with an AR(6) and seasonal MA(1) noise with expression:* $(1 - \phi₁B - \phi₂B^{2} - ... - \phi₆B⁶)(yₜ - yₜ₋₁₂) = \varepsilonₜ + \Theta₁ \varepsilonₜ₋₁₂$*”. (lines* ***60-63****.)*

*Additional notes/remarks*

- *The final model, once validated, was chosen by both adequacy (AIC/BIC) and prediction ability measures (MAPE, ML) which are summarized and reported in Table 2; see also Figures 2- 4.*
- *For predictive performance, we compared observed and predicted data in 2021 for the hepatitis data via the MAPE and ML metrics (see Table 2 and Figs. 2 and 3 corresponding to global CHB data and HCC subset).*
- *Regarding the forecasted estimate of the outcome measures and corresponding 95% confidence intervals for 2022, we present Figure 4 that shows point estimates and 95% CI of the outcome series for year 2022.*

5) **Methods:** There should be a section titled outcome measures (primary outcome and secondary measures) and predictors, including the time scale of the observations, with clearly defined measurement scales of the variables (count/discrete, continuous, binary, multinomial, ordinal) to understand the choice of statistical models. For instance, the outcome measure “hospital discharges” is it the number of monthly hospital discharges (count) or the percentage of hospital discharges per month (continuous)? Details are missing and must be provided

*We incorporated the suggested new section with the suggested content aspects. Specifically, we included the section:* ***Outcome measures*** *and paragraph: “The primary outcome time series is the global CHB number of monthly hospital discharges. The analysis of secondary but relevant HCC and cirrhosis subsets data related to CHB are also explored.* *The global chronic hepatitis B (CHB) related workload values range from 10 to 55 monthly discharges, while hepatitis B related to HCC and cirrhosis range from 4 to 34 and 1 to 29 discharges, respectively.” (****lines 251-256)***

*The final phrase in* ***lines 258-260*** *was removed from here and content incorporated above.*

6) **Methods:** The statistical method is quite detailed, with key assumptions under ARIMA (seasonality, autocorrelation, outlier treatment, etc) tested, including model performance measures such as AIC, BIC, MAPE, which was a plus. It looks great. However, authors must specify the functional form of the best model in the manuscript to ensure reproducibility of the results.

It is also not quite clear which distributional functional form and model was fitted. Poisson with ARIMA based model for count outcomes, OLS ARIMA based model etc. These details must be provided.

***Thanks.*** *As stated in the manuscript text, the chosen model for the main outcome measure, number of monthly CHB time series data and the subset HCC data, was the ARIMA(6,0,0)(0,1,1)_12 with outlier treatment.* *The functional form of this ARIMA model* *captures the series autoregressive dynamics with an AR(6) and seasonal MA(1) noise with expression:*

$(1 - \phi₁B - \phi₂B^{2} - ... - \phi₆B⁶)(yₜ - yₜ₋₁₂) = \varepsilonₜ + \Theta₁ \varepsilonₜ₋₁₂$*.* ***(lines 266-269)***

***This content was also incorporated in the abstract.***

7) **Results:** All the model‐based graph must show the observed trend (actual data used), predicted trend (based on the model) between 2017‐2021 and the forecasted (2021‐2022). All on one graph. This will help gauge the model predictive performance prior to the forecasting with diﬀerent color shade. It is so diﬃcult to see the graph. Kindly look at the resolution

***You are right, thank you.*** *Following your comments, we not only improved the resolution and aesthetics of all four figures but also slightly modified the corresponding captions for clarity and completeness. Additionally, please note that the two subfigures in the new Figure 4 have been arranged vertically, instead of horizontally as before.*

***Regarding the specific graph comments:***

- Figs. 2 and 3 show observed vs predicted (with 95%CI) discharge values for year 2021, based on the chosen ARIMAX model fitted initially with 2005-2020 train data. This allowed us to obtain the predictive performance of both, classic and ARIMAX extension (which includes outlier treatment).
- Forecasts for 12 months-horizon (year 2022) are displayed in Fig. 4. Note that, for each time series, a visual forecasting comparison can be done between the classic ARIMA and ARIMAX extension (dealing with outlier treatment).

**REVIEWER #2**

Dear Reviewer,

First of all, we would like to thank you for your valuable comments and suggestions, which have greatly helped us to improve the manuscript. We have incorporated all your recommendations into the new version of the document and highlighted them in red for easy identification. Below, we will respond to all of your comments. We will use an italic font for this purpose.

**Reviewer #2 comments**

The article is relevant, pertinent, and well written. However, I would like to make a specific comment regarding Table 1. In the section showing the diﬀerent codes for liver disease, I do not see an appropriate correlation between the ICD‐9 and ICD‐10 codes.

1. ‐ in the table, you present a generic code such as 571, and below it you list other, more specific terms. I assume that the codes actually used are the specific ones (571.XX) and not the generic 571.

*Yes, the codes that have been used are the specific ones, the generic code has been deleted from the table.*

2. ‐ the code 0.70 does not appear in the coding webpage of the Spanish Ministry of Health. htt[ps://w](http://www.eciemaps.sanidad.gob.es/browser/index_9_mc)ww[.eciemaps.sanidad.gob.es/br](http://www.eciemaps.sanidad.gob.es/browser/index_9_mc)ows[er/index_9_mc](http://www.eciemaps.sanidad.gob.es/browser/index_9_mc)

*You are right, there is a mistake and it has been removed from the table.*

3. ‐When comparing the ICD‐9 and ICD‐10 correspondence, they do not seem to match properly. htt[ps://w](http://www.eciemaps.sanidad.gob.es/browser/index_9_mc)ww[.eciemaps.sanidad.gob.es/br](http://www.eciemaps.sanidad.gob.es/browser/index_9_mc)ows[er/index_9_mc](http://www.eciemaps.sanidad.gob.es/browser/index_9_mc) For example, after reviewing:
   574.40 corresponds to K73.9
   574.41 corresponds to K73.0

574.49 corresponds to K73.2 and K73.8

571.5 corresponds to K74.0, K74.69, and K74.60

571.8 corresponds to K76.0 and K76.89
571.9 corresponds to K74.1 and K76.9

155.0 corresponds to C22.0

3. ‐ In the ICD‐10 column does not reflect what would be expected according to the ICD‐9 codes, and in addition, there are other terms listed that are not present in ICD‐9. por example: K75.3.

Since this issue concerns the main aspect of the study—patients with chronic liver disease and neoplasia—I believe it should be clarified and redefined with precision.

*Yes, we have remade the table. The codes listed on Table 1 have been corrected so that they correspond to those concerning patients discharged , no comparison has been carried out as to the two diferent codification.*

4.‐ I am not sure whether it is relevant for your study, but I also do not see the ICD‐9 and ICD‐10 codes for hepatitis B. If these were part of your analysis, they should be

explicitly included.

*You are right, these codes are specified in the methods section: “* *and the following secondary diagnoses of hepatitis B were used: 070.32 and 070.33 in ICD-9 and B18.0 and B18.2 in ICD-10”*
