# Supplementary material for: Modelling Hepatitis B virus related hospital discharges in Spain: ARIMAX based liver disease forecasting tool for hospital workload and mortality progression
Source: PLoS One. 2026 Jun 23;21(6):e0329751. doi: 10.1371/journal.pone.0329751 (PMC13289951; doi:10.1371/journal.pone.0329751)
Supplement: Attachment — Submitted filename: reply_to_editor_reviwer.docx [file pone.0329751.s003.docx]

### Rebuttal Comment to Editor’s query

After evaluating Revision 1, I am satisfied that most of the reviewers’ concerns have been adequately addressed. However, one essential methodological point remains unresolved. Reviewer 1 requested clarification regarding the **distributional functional form and the specific model fitted** in your ARIMAX framework. In particular, the reviewer noted, “It is also not quite clear which distributional functional form and model was fitted. Poisson with ARIMA-based model for count outcomes, OLS ARIMA-based model, etc. These details must be provided.”  This information is critical for ensuring transparency, reproducibility, and appropriate interpretation of your modelling approach. To proceed toward acceptance, please revise the manuscript to:

- Explicitly state the distributional assumptions underlying the model(s) used.
- Clarify whether the ARIMAX specification was implemented using a Poisson, quasi-Poisson, negative binomial, OLS, or other framework.
- Provide sufficient methodological detail for readers to understand and reproduce the modelling strategy, including any justification for the chosen distributional form.

Given that all other issues have been satisfactorily addressed, this clarification constitutes a **minor revision**. No additional reviewer input will be required once this point is resolved.

### Dear editor:

We thank you and the reviewer for this important methodological concern about the distributional assumptions of the final fitted model. Thus, as requested, we have updated the 'Time series analysis' section (Lines 211–224) to explicitly state that a Gaussian ARIMAX framework was used; entry in lines 54-55 was also corrected.

We have clarified that the model assumes a Normal distribution for the error terms and that this assumption was validated via residual diagnostics, confirming that a discrete distributional form (e.g., Poisson) was not required for this dataset. We marked in blue the new entries.

### Revised Manuscript Text (Abstract 54–55)

… with an AR(6) and seasonal MA(1) noise with expression: (1 − 𝜙₁ 𝐵 − 𝜙₂ 𝐵² − . . . − 𝜙₆ 𝐵⁶) (𝑦ₜ − 𝑦ₜ₋₁₂) = 𝜀ₜ + 𝛩₁ 𝜀ₜ₋₁₂ with 𝜀 ~N(0, σ²)*.*

### Revised Manuscript Text (Lines 211–224)

The behavior of hepatitis B virus time series analysis from 2005–2020 was studied and used to identify and fit different plausible ARIMA models. Then, known data from 2021 was used for model testing and finally forecast was performed for 2022. The best fit was obtained with Model ARIMA (6, 0, 0) (0, 1, 1)_12, which incorporates information of six previous months and one seasonal innovation term (Figure 2). This model was implemented using a Gaussian distributional functional form, where the parameters were estimated via Maximum Likelihood Estimation (MLE). While the data consists of counts, the volume of monthly cases was sufficient to support a continuous approximation; the assumption that the residuals (𝜀ₜ) follow a Normal distribution with constant variance was validated through post-estimation diagnostic testing of the residuals. The functional form of this model captures the series autoregressive dynamics with an AR(6) and a seasonal noise with seasonal MA(1) with expression: (1 − 𝜙₁𝐵 − 𝜙₂𝐵² − . . . − 𝜙₆𝐵⁶)(𝑦ₜ − 𝑦ₜ₋₁₂) = 𝜀ₜ +𝛩₁ 𝜀ₜ₋₁₂ with 𝜀 ~N(0, σ²).
