# Supplementary material for: Modelling Hepatitis B virus related hospital discharges in Spain: ARIMAX based liver disease forecasting tool for hospital workload and mortality progression
Source: PLoS One. 2026 Jun 23;21(6):e0329751. doi: 10.1371/journal.pone.0329751 (PMC13289951; doi:10.1371/journal.pone.0329751)
Supplement: Attachment — Submitted filename: Response_to_reviewers_auresp_3.docx [file pone.0329751.s004.docx]

**Dear Editor and Reviewer 1,**

We sincerely thank you for the opportunity to further clarify our methodological approach. We appreciate Reviewer 1’s detailed and insightful comments regarding the structural properties of count data, including non-negativity, discreteness, and multiplicative dynamics. We fully agree that a naive application of Gaussian ARIMA to raw, low level counts is inappropriate.

However, we wish to clarify that our modelling approach was not conducted on raw counts, but within a **Variance-Stabilizing Transformation (VST)** framework specifically designed to map discrete count processes into a Gaussian-compliant space. While this step was implemented in the original analysis, we acknowledge it was not explicitly documented; we have now corrected this in the revised manuscript.

We provide the following rigorous statistical justification for the use of the approximate Gaussian ARIMAX methodology:

1. **Variance-stabilizing transformation (VST):** To address the issues of heteroscedasticity and the multiplicative dynamics inherent in count data, we applied a **square-root transformation** (Box–Cox with λ=0.5) prior to estimation. As established by **Bartlett (1936)**, the square-root transformation is the optimal VST for Poisson-distributed data, as it transforms the mean-variance relationship (where Var(Y)=μ) into an approximately constant variance (Var($\sqrt{\boldsymbol{Y}}$) $\approx0$.25). Lines 167-172

This transformation specifically addresses the Reviewer’s concerns by: **linearizing multiplicative dynamics, because i**t mitigates the mismatch between the additive structure of ARIMAX and the multiplicative nature of counts, and by **normalizing the error structure:** It allows the use of Gaussian Likelihood while maintaining consistency with the underlying count process **(Shumway and Stoffer, 2016)**.

2. **Suitability of the continuous approximation for count data:** While hospital discharges are discrete, our aggregated monthly counts range from 10 to 55. For a Poisson process P(λ), the distribution approaches normality as λ increases. It is a well-accepted convention in time series analysis that when λ≥10, the loss of information incurred by a continuous approximation is negligible (**Box et al., 2005; Box et al., 2008**). In our dataset, the mean is sufficiently far from the zero boundary to treat the transformed series as a continuous Gaussian process without risking "boundary effects." Lines 225-233
3. **Consistency via quasi-maximum likelihood estimation (QMLE):** Parameter estimation was conducted using a Gaussian likelihood function. Under standard regularity conditions, this approach yields consistent parameter estimates for ARMA-type models even when the true conditional distribution deviates from Gaussianity, provided the conditional mean and variance are correctly specified and the innovations have finite variance **(Hamilton, 1994; Tsay, 2010).** Lines 335-340
4. **Residual diagnostics:** As noted in our previous review, post-estimation graphical tools and diagnostic testing, including assessments of autocorrelation (Box–Ljung test), homoscedasticity, and normality, indicated that the residuals behave as Gaussian white noise. These results support the adequacy of the approximate Gaussian ARIMAX structure in successfully capturing the temporal dynamics of the series. Lines 235-237
5. **Non-Negativity of predictions:** A primary concern raised was the possibility of negative predictions. We have verified that: all in sample fits, out-of-sample predictions and forecasts were strictly non-negative. Given the mean (μ) and the estimated variance (σ^2) of our transformed series, the probability of the model generating a negative value is statistically negligible (the zero boundary lies far away from the mean). This indicates that the model respected the natural bounds of the data despite not imposing an explicit non-negativity constraint. Lines 280-287
6. **Predictive performance:** The approximate ARIMAX model achieved good predictive accuracy (MAPE = 13.9%), providing empirical evidence that the Gaussian approximation, when combined with a VST, yields a robust representation of the system's underlying data-generating process. Lines 280-287

**Conclusion:**

In light of these points, we believe the approximate Gaussian $\sqrt{\boldsymbol{Y}}$**-ARIMAX** approach is not only appropriate but provides a parsimonious and rigorously justified alternative to specialized count models (which can often suffer from convergence issues or over-parameterization in small-to-moderate sample sizes). Thus, as our results confirm that the transformation effectively addressed the discreteness and heteroscedasticity of the series, this approach stands as a statistically sound and parsimonious methodology for the research objective.

**Specific Manuscript Edits under this revision**

**Note:** *We have updated (red font) the* ***Material and Methods*** *and* ***the Discussion*** *to explicitly detail the statistical rationale for the square root transformation and the adequacy of the Gaussian approximation for this study.* Additional clarifying review inputs are clearly reflected in red font.

**Author Contributions:**

**Abstract**: (methods y results)

**Material and methods** section: (statistical methodology)

**Results:** (, time series anal:ysis,)

**Discussion**

1. **Mention the Transformation:** "To account for the discrete, non-negative nature of hospital discharge counts and to stabilize the variance, a square-root transformation was applied to the dependent variable ($y_t' = \sqrt{y_t}$) prior to ARIMAX modeling (Bartlett, 1936)."
2. **Mention the Boundary:** "Given that monthly counts exceeded 10 in all periods, a Gaussian approximation was deemed appropriate (Box et al., 2015)."
3. **Refine the Residuals Section:** "Residual diagnostics confirmed the adequacy of this transformation, showing no evidence of heteroscedasticity or non-normality."

**References added or updated**

Bartlett, M.S. (1936). The Square Root Transformation in Analysis of Variance. Supplement to the Journal of the Royal Statistical Society, *3*(1), 68–78. <https://doi-org.recursos.biblioteca.upc.edu/10.2307/2983678>

Box GEP, Hunter JS, Hunter WG (2005) Statistics for experimenters. 2nd ed. Hoboken (NJ): John Wiley & Sons.

Box GEP, Jenkins GM, Reinsel GC (2008) Time series analysis: forecasting and control. 4th ed. Hoboken (NJ): John Wiley & Sons.

Shumway RH, Stoffer DS (2016) Time series analysis and its applications: with R examples. Available from: <http://www.stat.ucla.edu/~frederic/415/S23/tsa4.pdf>

Hamilton JD (1994) *Time series analysis*. Princeton (NJ): Princeton University Press.

Tsay RS (2010) *Analysis of financial time series*. 3rd ed. Hoboken (NJ): John Wiley & Sons.
