# Supplementary material for: Modelling Hepatitis B virus related hospital discharges in Spain: ARIMAX based liver disease forecasting tool for hospital workload and mortality progression
Source: PLoS One. 2026 Jun 23;21(6):e0329751. doi: 10.1371/journal.pone.0329751 (PMC13289951; doi:10.1371/journal.pone.0329751)
Supplement: Peer review history

# Peer review: Decision Letter 0

**DOI:** 10.1371/journal.pone.0329751.r001

**Authors:** Yordanis Enríquez Canto

22 Sep 2025

PONE-D-25-37298

Modelling Hepatitis B virus related hospital discharges in Spain: ARIMAX based liver disease forecasting tool for hospital workload and mortality progression

PLOS ONE

Dear Dr. Torner,

Thank you for submitting your manuscript to PLOS ONE. After careful consideration, we feel that it has merit but does not fully meet PLOS ONE’s publication criteria as it currently stands. Therefore, we invite you to submit a revised version of the manuscript that addresses the points raised during the review process.

Here’s a concise set of central points to address for greater methodological rigor:

- Correct and validate Table 1 ICD mappings
  Use specific ICD-9 subcodes (571.xx etc.), not generic headers.
- Remove invalid codes (e.g., 0.70) and reconcile all ICD-9 ↔ ICD-10 correspondences using the Spanish Ministry of Health browser.
- Ensure one-to-many mappings are accurately reflected; remove ICD-10 terms without ICD-9 counterparts unless justified.
- Explicitly include ICD-9/ICD-10 codes for hepatitis B, HCC, cirrhosis, and chronic liver disease.
- Enhance the Abstract by including quantitative outputs from the model.
- Report CIs for total discharges and proportions (HCC, cirrhosis).
- Replace methodological statements with key modelling results: best model(s), 2021 observed vs. predicted, and 2022 forecasts with CIs.
- Fix incomplete statement on cirrhosis model.
- Clarify outcomes, predictors, and time scale
  - Add a dedicated section defining primary/secondary outcomes and predictors.
  - Specify measurement scales (counts vs. percentages; monthly vs. other time unit).
- Specify the modelling framework for reproducibility
  Please specify the exact model class (e.g., ARIMAX with Poisson, OLS, or another link), the distributional assumptions, and the functional form including orders, seasonal components, and exogenous variables.
- Report selection criteria and performance metrics (AIC, BIC, MAPE) for the chosen model(s).
- Present integrated performance/forecast figures
  For each outcome, show observed (2017–2021), in-sample fitted/predicted (2017–2021), and forecast (2021–2022) on one high-resolution plot with clear legends and color-coding.

Addressing these will materially improve validity, transparency, and reproducibility.

Please submit your revised manuscript by Nov 06 2025 11:59PM. If you will need more time than this to complete your revisions, please reply to this message or contact the journal office at plosone@plos.org. When you’re ready to submit your revision, log on to https://www.editorialmanager.com/pone/ and select the ‘Submissions Needing Revision’ folder to locate your manuscript file.

Please include the following items when submitting your revised manuscript:

- A rebuttal letter that responds to each point raised by the academic editor and reviewer(s). You should upload this letter as a separate file labeled ‘Response to Reviewers’.
- A marked-up copy of your manuscript that highlights changes made to the original version. You should upload this as a separate file labeled ‘Revised Manuscript with Track Changes’.
- An unmarked version of your revised paper without tracked changes. You should upload this as a separate file labeled ‘Manuscript’.

If you would like to make changes to your financial disclosure, please include your updated statement in your cover letter. Guidelines for resubmitting your figure files are available below the reviewer comments at the end of this letter.

If applicable, we recommend that you deposit your laboratory protocols in protocols.io to enhance the reproducibility of your results. Protocols.io assigns your protocol its own identifier (DOI) so that it can be cited independently in the future. For instructions see: https://journals.plos.org/plosone/s/submission-guidelines#loc-laboratory-protocols. Additionally, PLOS ONE offers an option for publishing peer-reviewed Lab Protocol articles, which describe protocols hosted on protocols.io. Read more information on sharing protocols at https://plos.org/protocols?utm_medium=editorial-email&utm_source=authorletters&utm_campaign=protocols.

We look forward to receiving your revised manuscript.

Kind regards,

Yordanis Enríquez Canto, Ph.D.

Academic Editor

PLOS ONE

Journal Requirements:

When submitting your revision, we need you to address these additional requirements.

1. Please ensure that your manuscript meets PLOS ONE’s style requirements, including those for file naming. The PLOS ONE style templates can be found at

https://journals.plos.org/plosone/s/file?id=wjVg/PLOSOne_formatting_sample_main_body.pdf and

https://journals.plos.org/plosone/s/file?id=ba62/PLOSOne_formatting_sample_title_authors_affiliations.pdf

2. Please note that PLOS One has specific guidelines on code sharing for submissions in which author-generated code underpins the findings in the manuscript. In these cases, we expect all author-generated code to be made available without restrictions upon publication of the work. Please review our guidelines at https://journals.plos.org/plosone/s/materials-and-software-sharing#loc-sharing-code and ensure that your code is shared in a way that follows best practice and facilitates reproducibility and reuse.

3. Thank you for stating the following financial disclosure:

Programme of Prevention, Surveillance, and Control of Transmissible Diseases (PREVICET), CIBER de Epidemiología y Salud Pública (CIBERESP), Instituto de Salud Carlos III, Madrid

Please state what role the funders took in the study. If the funders had no role, please state: "The funders had no role in study design, data collection and analysis, decision to publish, or preparation of the manuscript."

If this statement is not correct you must amend it as needed.

Please include this amended Role of Funder statement in your cover letter; we will change the online submission form on your behalf.

4. Please remove your figures from within your manuscript file, leaving only the individual TIFF/EPS image files, uploaded separately. These will be automatically included in the reviewers’ PDF.

5. Please include your tables as part of your main manuscript and remove the individual files. Please note that supplementary tables (should remain/ be uploaded) as separate "supporting information" files

6. Please include captions for your Supporting Information files at the end of your manuscript, and update any in-text citations to match accordingly. Please see our Supporting Information guidelines for more information: http://journals.plos.org/plosone/s/supporting-information.

7. If the reviewer comments include a recommendation to cite specific previously published works, please review and evaluate these publications to determine whether they are relevant and should be cited. There is no requirement to cite these works unless the editor has indicated otherwise.

[Note: HTML markup is below. Please do not edit.]

Reviewers’ comments:

Reviewer's Responses to Questions

**Comments to the Author**

1. Is the manuscript technically sound, and do the data support the conclusions?

The manuscript must describe a technically sound piece of scientific research with data that supports the conclusions. Experiments must have been conducted rigorously, with appropriate controls, replication, and sample sizes. The conclusions must be drawn appropriately based on the data presented.

Reviewer #1: Yes

Reviewer #2: Yes

**********

2. Has the statistical analysis been performed appropriately and rigorously?

Reviewer #1: Yes

Reviewer #2: I Don't Know

**********

3. Have the authors made all data underlying the findings in their manuscript fully available?

The PLOS Data policy requires authors to make all data underlying the findings described in their manuscript fully available without restriction, with rare exception (please refer to the Data Availability Statement in the manuscript PDF file). The data should be provided as part of the manuscript or its supporting information, or deposited to a public repository. For example, in addition to summary statistics, the data points behind means, medians and variance measures should be available. If there are restrictions on publicly sharing data—e.g. participant privacy or use of data from a third party—those must be specified.

Reviewer #1: Yes

Reviewer #2: Yes

**********

4. Is the manuscript presented in an intelligible fashion and written in standard English?

PLOS ONE does not copyedit accepted manuscripts, so the language in submitted articles must be clear, correct, and unambiguous. Any typographical or grammatical errors should be corrected at revision, so please note any specific errors here.

Reviewer #1: Yes

Reviewer #2: Yes

**********

5. Review Comments to the Author

Please use the space provided to explain your answers to the questions above. You may also include additional comments for the author, including concerns about dual publication, research ethics, or publication ethics. (Please upload your review as an attachment if it exceeds 20,000 characters)

Reviewer #1: Dear Editor,

Thank you for the opportunity to review the manuscript titled “Modelling Hepatitis B virus related hospital discharges in Spain: ARIMAX based liver disease forecasting tool for hospital workload and mortality progression”

Here are my comments

Abstract: Kindly report the confidence interval for the total number of discharges, percentage attributed to HCC, and cirrhosis to provide a better narrative of what is happening in the target population beyond the sample.

Abstract: This statement is not result but methodology: “CHB time series analysis with data from 2005-2020 was used to fit plausible ARIMA models, followed by testing with known 2021 data to obtain 2022 forecasts”

Abstract: The best fit was obtained with the model, except that cirrhosis should be revised. It sounds incomplete

The results presented in the abstract section are just descriptive summary measures, and nothing about the results from the actual modelling study. Kindly present the key results of the modelling study. What was the best predictive model based on the model performance measures? How did the observed data in 2021 compare with the predicted data from the model in 2021? What was the forecasted estimate of the outcome measure and corresponding confidence interval in 2022

Methods

There should be a section titled outcome measures (primary outcome and secondary measures) and predictors, including the time scale of the observations, with clearly defined measurement scales of the variables (count/discrete, continuous, binary, multinomial, ordinal) to understand the choice of statistical models. For instance, the outcome measure “hospital discharges” is it the number of monthly hospital discharges (count) or the percentage of hospital discharges per month (continuous)? Details are missing and must be provided

The statistical method is quite detailed, with key assumptions under ARIMA (seasonality, autocorrelation, outlier treatment, etc) tested, including model performance measures such as AIC, BIC, MAPE, which was a plus. It looks great. However, authors must specify the functional form of the best model in the manuscript to ensure reproducibility of the results.

It is also not quite clear which distributional functional form and model was fitted. Poisson with ARIMA based model for count outcomes, OLS ARIMA based model etc. These details must be provided.

Results

All the model-based graph must show the observed trend (actual data used), predicted trend (based on the model) between 2017-2021 and the forecasted (2021-2022). All on one graph. This will help gauge the model predictive performance prior to the forecasting with different color shade. It is so difficult to see the graph. Kindly look at the resolution

Reviewer #2: The article is relevant, pertinent, and well written. However, I would like to make a specific comment regarding Table 1. In the section showing the different codes for liver disease, I do not see an appropriate correlation between the ICD-9 and ICD-10 codes.

1.- in the table, you present a generic code such as 571, and below it you list other, more specific terms. I assume that the codes actually used are the specific ones (571.XX) and not the generic 571.

2.- the code 0.70 does not appear in the coding webpage of the Spanish Ministry of Health. https://www.eciemaps.sanidad.gob.es/browser/index_9_mc

3.-When comparing the ICD-9 and ICD-10 correspondence, they do not seem to match properly. https://www.eciemaps.sanidad.gob.es/browser/index_9_mc For example, after reviewing:

574.40 corresponds to K73.9

574.41 corresponds to K73.0

574.49 corresponds to K73.2 and K73.8

571.5 corresponds to K74.0, K74.69, and K74.60

571.8 corresponds to K76.0 and K76.89

571.9 corresponds to K74.1 and K76.9

155.0 corresponds to C22.0

3.- In the ICD-10 column does not reflect what would be expected according to the ICD-9 codes, and in addition, there are other terms listed that are not present in ICD-9. por example: K75.3

Since this issue concerns the main aspect of the study—patients with chronic liver disease and neoplasia—I believe it should be clarified and redefined with precision.

4.- I am not sure whether it is relevant for your study, but I also do not see the ICD-9 and ICD-10 codes for hepatitis B. If these were part of your analysis, they should be explicitly included.

**********

6. PLOS authors have the option to publish the peer review history of their article (what does this mean?). If published, this will include your full peer review and any attached files.

If you choose “no”, your identity will remain anonymous but your review may still be made public.

**Do you want your identity to be public for this peer review?**  For information about this choice, including consent withdrawal, please see our Privacy Policy.

Reviewer #1: No

Reviewer #2: No

**********

[NOTE: If reviewer comments were submitted as an attachment file, they will be attached to this email and accessible via the submission site. Please log into your account, locate the manuscript record, and check for the action link "View Attachments". If this link does not appear, there are no attachment files.]

While revising your submission, please upload your figure files to the Preflight Analysis and Conversion Engine (PACE) digital diagnostic tool, https://pacev2.apexcovantage.com/. PACE helps ensure that figures meet PLOS requirements. To use PACE, you must first register as a user. Registration is free. Then, login and navigate to the UPLOAD tab, where you will find detailed instructions on how to use the tool. If you encounter any issues or have any questions when using PACE, please email PLOS at figures@plos.org. Please note that Supporting Information files do not need this step.

# Author comment: Author response to Decision Letter 1

**DOI:** 10.1371/journal.pone.0329751.r002

18 Dec 2025

Manuscript title: “Modelling Hepatitis B virus related hospital discharges in Spain: ARIMAX based liver disease forecasting tool for hospital workload and mortality progression”

Response to Editor and Reviewers

Dear Editor:

We greatly appreciate your interest in our work and the opportunity to improve the manuscript. We have revised the paper according to the reviewers’ suggestions, with all changes highlighted in red in the updated version. We have also prepared a detailed response letter in which each reviewer comment is reproduced, followed by our response in italic text. We also thank you for your feedback and help in this process.

REVIEWER #1

Dear Reviewer,

First of all, we would like to thank you for your valuable comments and suggestions, which have greatly helped us to improve the manuscript. We have incorporated all your recommendations into the new version of the document and highlighted them in red for easy identification. Below, we will respond to all of your comments. We will use an italic font for this purpose.

Additional notes/remarks:

Below, we addressed all central points made by two reviewers and that you kindly summarized for us.

As required, all plots were removed from within the manuscript. See attached .tiff files and corresponding captions (List of Figures and captionsLA.docx).

We created a “Revised Manuscript with Track Changes.docx” document with red-marks to be uploaded separately. Also, we attach the unmark version in the “Manuscript.docx” File.

Reviewer #1 comments

Here are my comments

Abstract: Kindly report the confidence interval for the total number of discharges, percentage attributed to HCC, and cirrhosis to provide a better narrative of what is happening in the target population beyond the sample.

The percentage attributed to HCC, and cirrhosis is provided in line 53 of the abstract. To provide a better narrative of what is happening in the target population beyond the sample, we only aim to provide point predictions (and corresponding 95% CI to quantify uncertainty) via the classic ARIMA and ARIMAX extension method. However, although we do not make any inference on discharge data metrics, following your comment to provide a better general description of the three times series, we added the phrase: “The global chronic hepatitis B (CHB) related workload values range from 10 to 55 monthly discharges, while hepatitis B related to HCC and cirrhosis range from 4 to 34 and 1 to 29 discharges, respectively” (lines 56-58; see Fig. 1 also).

Abstract: This statement is not result but methodology: “CHB time series analysis with data from 2005‐2020 was used to fit plausible ARIMA models, followed by testing with known 2021 data to obtain 2022 forecasts”

You are right, thank you. We removed such statement from the abstract and checked that this information/content was already incorporated in section “Materials and methods”.

Abstract: The best fit was obtained with the model, except that cirrhosis should be revised. It sounds incomplete

You are right, the sentence was incomplete. We modified it to be complete and more clearly stated, by adding in lines x-x, the sentence: “The best fit and 2022 forecasts found for CHB and HCC time series was obtained with model ARIMAX (6,0,0) (0,1,1)_12.” (lines 58-59).

Abstract: The results presented in the abstract section are just descriptive summary measures, and nothing about the results from the actual modelling study. Kindly present the key results of the modelling study. What was the best predictive model based on the model performance measures? How did the observed data in 2021 compare with the predicted data from the model in 2021? What was the forecasted estimate of the outcome measure and corresponding confidence interval in 2022 Methods

Now, apart from key results of the modelling and forecasting study provided before (following your 3rd-comment), we added a sentence regarding the functional form of the chosen model: “This model after treating outliers, removes the seasonal patterns through seasonal differencing and captures the series autoregressive dynamics with an AR(6) and seasonal MA(1) noise with expression: (1 - ϕ₁B - ϕ₂B² - ...- ϕ₆B⁶)(yₜ - yₜ1₁₂) = εₜ + Θ₁ εₜ₋₁₂”. (lines 60-63.)

Additional notes/remarks

The final model, once validated, was chosen by both adequacy (AIC/BIC) and prediction ability measures (MAPE, ML) which are summarized and reported in Table 2; see also Figures 2- 4.

For predictive performance, we compared observed and predicted data in 2021 for the hepatitis data via the MAPE and ML metrics (see Table 2 and Figs. 2 and 3 corresponding to global CHB data and HCC subset).

Regarding the forecasted estimate of the outcome measures and corresponding 95% confidence intervals for 2022, we present Figure 4 that shows point estimates and 95% CI of the outcome series for year 2022.

Methods: There should be a section titled outcome measures (primary outcome and secondary measures) and predictors, including the time scale of the observations, with clearly defined measurement scales of the variables (count/discrete, continuous, binary, multinomial, ordinal) to understand the choice of statistical models. For instance, the outcome measure “hospital discharges” is it the number of monthly hospital discharges (count) or the percentage of hospital discharges per month (continuous)? Details are missing and must be provided

We incorporated the suggested new section with the suggested content aspects. Specifically, we included the section: Outcome measures and paragraph: “The primary outcome time series is the global CHB number of monthly hospital discharges. The analysis of secondary but relevant HCC and cirrhosis subsets data related to CHB are also explored. The global chronic hepatitis B (CHB) related workload values range from 10 to 55 monthly discharges, while hepatitis B related to HCC and cirrhosis range from 4 to 34 and 1 to 29 discharges, respectively.” (lines 251-256)

The final phrase in lines 258-260 was removed from here and content incorporated above.

Methods: The statistical method is quite detailed, with key assumptions under ARIMA (seasonality, autocorrelation, outlier treatment, etc) tested, including model performance measures such as AIC, BIC, MAPE, which was a plus. It looks great. However, authors must specify the functional form of the best model in the manuscript to ensure reproducibility of the results.

It is also not quite clear which distributional functional form and model was fitted. Poisson with ARIMA based model for count outcomes, OLS ARIMA based model etc. These details must be provided.

Thanks. As stated in the manuscript text, the chosen model for the main outcome measure, number of monthly CHB time series data and the subset HCC data, was the ARIMA(6,0,0)(0,1,1)_12 with outlier treatment. The functional form of this ARIMA model captures the series autoregressive dynamics with an AR(6) and seasonal MA(1) noise with expression:

(1 - ϕ₁B - ϕ₂B² - ...- ϕ₆B⁶)(yₜ - yₜ₋₁₂) = εₜ + Θ₁ εₜ₋₁₂. (lines 266-269)

This content was also incorporated in the abstract.

Results: All the model‐based graph must show the observed trend (actual data used), predicted trend (based on the model) between 2017‐2021 and the forecasted (2021‐2022). All on one graph. This will help gauge the model predictive performance prior to the forecasting with diﬀerent color shade. It is so diﬃcult to see the graph. Kindly look at the resolution

You are right, thank you. Following your comments, we not only improved the resolution and aesthetics of all four figures but also slightly modified the corresponding captions for clarity and completeness. Additionally, please note that the two subfigures in the new Figure 4 have been arranged vertically, instead of horizontally as before.

Regarding the specific graph comments:

Figs. 2 and 3 show observed vs predicted (with 95%CI) discharge values for year 2021, based on the chosen ARIMAX model fitted initially with 2005-2020 train data. This allowed us to obtain the predictive performance of both, classic and ARIMAX extension (which includes outlier treatment).

Forecasts for 12 months-horizon (year 2022) are displayed in Fig. 4. Note that, for each time series, a visual forecasting comparison can be done between the classic ARIMA and ARIMAX extension (dealing with outlier treatment).

REVIEWER #2

Dear Reviewer,

First of all, we would like to thank you for your valuable comments and suggestions, which have greatly helped us to improve the manuscript. We have incorporated all your recommendations into the new version of the document and highlighted them in red for easy identification. Below, we will respond to all of your comments. We will use an italic font for this purpose.

Reviewer #2 comments

The article is relevant, pertinent, and well written. However, I would like to make a specific comment regarding Table 1. In the section showing the diﬀerent codes for liver disease, I do not see an appropriate correlation between the ICD‐9 and ICD‐10 codes.

‐ in the table, you present a generic code such as 571, and below it you list other, more specific terms. I assume that the codes actually used are the specific ones (571.XX) and not the generic 571.

Yes, the codes that have been used are the specific ones, the generic code has been deleted from the table.

‐ the code 0.70 does not appear in the coding webpage of the Spanish Ministry of Health. https://www.eciemaps.sanidad.gob.es/browser/index_9_mc

You are right, there is a mistake and it has been removed from the table.

‐When comparing the ICD‐9 and ICD‐10 correspondence, they do not seem to match properly. https://www.eciemaps.sanidad.gob.es/browser/index_9_mc For example, after reviewing:

corresponds to K73.9

corresponds to K73.0

574.49 corresponds to K73.2 and K73.8

571.5 corresponds to K74.0, K74.69, and K74.60

corresponds to K76.0 and K76.89

corresponds to K74.1 and K76.9

155.0 corresponds to C22.0

‐ In the ICD‐10 column does not reflect what would be expected according to the ICD‐9 codes, and in addition, there are other terms listed that are not present in ICD‐9. por example: K75.3.

Since this issue concerns the main aspect of the study—patients with chronic liver disease and neoplasia—I believe it should be clarified and redefined with precision.

Yes, we have remade the table. The codes listed on Table 1 have been corrected so that they correspond to those concerning patients discharged , no comparison has been carried out as to the two diferent codification.

4.‐ I am not sure whether it is relevant for your study, but I also do not see the ICD‐9 and ICD‐10 codes for hepatitis B. If these were part of your analysis, they should be

explicitly included.

You are right, these codes are specified in the methods section: “ and the following secondary diagnoses of hepatitis B were used: 070.32 and 070.33 in ICD-9 and B18.0 and B18.2 in ICD-10”

Attachment Submitted filename: Response to Reviewers.docx

# Peer review: Decision Letter 1

**DOI:** 10.1371/journal.pone.0329751.r003

**Authors:** Yordanis Enríquez Canto, Yordanis Enríquez Canto

29 Jan 2026

PONE-D-25-37298R1

Modelling Hepatitis B virus related hospital discharges in Spain: ARIMAX based liver disease forecasting tool for hospital workload and mortality progression

PLOS One

Dear Dr. Torner,

Thank you for submitting your manuscript to PLOS ONE. After careful consideration, we feel that it has merit but does not fully meet PLOS ONE’s publication criteria as it currently stands. Therefore, we invite you to submit a revised version of the manuscript that addresses the points raised during the review process.

==============================

After evaluating Revision 1, I am satisfied that most of the reviewers’ concerns have been adequately addressed. However, one essential methodological point remains unresolved. Reviewer 1 requested clarification regarding the **distributional functional form and the specific model fitted**  in your ARIMAX framework. In particular, the reviewer noted, “It is also not quite clear which distributional functional form and model was fitted. Poisson with ARIMA-based model for count outcomes, OLS ARIMA-based model, etc. These details must be provided.”  This information is critical for ensuring transparency, reproducibility, and appropriate interpretation of your modelling approach. To proceed toward acceptance, please revise the manuscript to:

- Explicitly state the distributional assumptions underlying the model(s) used.
- Clarify whether the ARIMAX specification was implemented using a Poisson, quasi-Poisson, negative binomial, OLS, or other framework.
- Provide sufficient methodological detail for readers to understand and reproduce the modelling strategy, including any justification for the chosen distributional form.

Given that all other issues have been satisfactorily addressed, this clarification constitutes a **minor revision** . No additional reviewer input will be required once this point is resolved.

==============================

Please submit your revised manuscript by Mar 15 2026 11:59PM. If you will need more time than this to complete your revisions, please reply to this message or contact the journal office at plosone@plos.org. When you're ready to submit your revision, log on to https://www.editorialmanager.com/pone/ and select the 'Submissions Needing Revision' folder to locate your manuscript file.

Please include the following items when submitting your revised manuscript:

- A letter that responds to each point raised by the academic editor and reviewer(s). You should upload this letter as a separate file labeled 'Response to Reviewers'.
- A marked-up copy of your manuscript that highlights changes made to the original version. You should upload this as a separate file labeled 'Revised Manuscript with Track Changes'.
- An unmarked version of your revised paper without tracked changes. You should upload this as a separate file labeled 'Manuscript'.

-->If you would like to make changes to your financial disclosure, please include your updated statement in your cover letter. Guidelines for resubmitting your figure files are available below the reviewer comments at the end of this letter.

If applicable, we recommend that you deposit your laboratory protocols in protocols.io to enhance the reproducibility of your results. Protocols.io assigns your protocol its own identifier (DOI) so that it can be cited independently in the future. For instructions see: https://journals.plos.org/plosone/s/submission-guidelines#loc-laboratory-protocols. Additionally, PLOS ONE offers an option for publishing peer-reviewed Lab Protocol articles, which describe protocols hosted on protocols.io. Read more information on sharing protocols at https://plos.org/protocols?utm_medium=editorial-email&utm_source=authorletters&utm_campaign=protocols.

We look forward to receiving your revised manuscript.

Kind regards,

Yordanis Enríquez Canto, Ph.D.

Academic Editor

PLOS One

**Journal Requirements:**

1. If the reviewer comments include a recommendation to cite specific previously published works, please review and evaluate these publications to determine whether they are relevant and should be cited. There is no requirement to cite these works unless the editor has indicated otherwise.

2. Please review your reference list to ensure that it is complete and correct. If you have cited papers that have been retracted, please include the rationale for doing so in the manuscript text, or remove these references and replace them with relevant current references. Any changes to the reference list should be mentioned in the rebuttal letter that accompanies your revised manuscript. If you need to cite a retracted article, indicate the article’s retracted status in the References list and also include a citation and full reference for the retraction notice.

[Note: HTML markup is below. Please do not edit.]

Reviewers' comments:

Reviewer's Responses to Questions

**Comments to the Author**

1. If the authors have adequately addressed your comments raised in a previous round of review and you feel that this manuscript is now acceptable for publication, you may indicate that here to bypass the “Comments to the Author” section, enter your conflict of interest statement in the “Confidential to Editor” section, and submit your "Accept" recommendation.

Reviewer #1: All comments have been addressed

**********

2. Is the manuscript technically sound, and do the data support the conclusions?

The manuscript must describe a technically sound piece of scientific research with data that supports the conclusions. Experiments must have been conducted rigorously, with appropriate controls, replication, and sample sizes. The conclusions must be drawn appropriately based on the data presented.

Reviewer #1: Yes

**********

3. Has the statistical analysis been performed appropriately and rigorously?

Reviewer #1: Yes

**********

4. Have the authors made all data underlying the findings in their manuscript fully available?

The PLOS Data policy requires authors to make all data underlying the findings described in their manuscript fully available without restriction, with rare exception (please refer to the Data Availability Statement in the manuscript PDF file). The data should be provided as part of the manuscript or its supporting information, or deposited to a public repository. For example, in addition to summary statistics, the data points behind means, medians and variance measures should be available. If there are restrictions on publicly sharing data—e.g. participant privacy or use of data from a third party—those must be specified.

Reviewer #1: Yes

**********

5. Is the manuscript presented in an intelligible fashion and written in standard English?

PLOS ONE does not copyedit accepted manuscripts, so the language in submitted articles must be clear, correct, and unambiguous. Any typographical or grammatical errors should be corrected at revision, so please note any specific errors here.

Reviewer #1: Yes

**********

6. Review Comments to the Author

Please use the space provided to explain your answers to the questions above. You may also include additional comments for the author, including concerns about dual publication, research ethics, or publication ethics. (Please upload your review as an attachment if it exceeds 20,000 characters)

**Reviewer #1:**  Most comments were addressed except this which is very important "

It is also not quite clear which distributional functional form and model was fitted. Poisson with ARIMA based model for count outcomes, OLS ARIMA based model etc. These details must be provided"

**********

7. PLOS authors have the option to publish the peer review history of their article (what does this mean?). If published, this will include your full peer review and any attached files.

If you choose “no”, your identity will remain anonymous but your review may still be made public.

**Do you want your identity to be public for this peer review?**  For information about this choice, including consent withdrawal, please see our Privacy Policy.

Reviewer #1: No

**********

[NOTE: If reviewer comments were submitted as an attachment file, they will be attached to this email and accessible via the submission site. Please log into your account, locate the manuscript record, and check for the action link "View Attachments". If this link does not appear, there are no attachment files.]

To ensure your figures meet our technical requirements, please review our figure guidelines: https://journals.plos.org/plosone/s/figures

You may also use PLOS’s free figure tool, NAAS, to help you prepare publication quality figures: https://journals.plos.org/plosone/s/figures#loc-tools-for-figure-preparation.

NAAS will assess whether your figures meet our technical requirements by comparing each figure against our figure specifications.

# Author comment: Author response to Decision Letter 2

**DOI:** 10.1371/journal.pone.0329751.r004

25 Feb 2026

Rebuttal Comment to Editor’s query

After evaluating Revision 1, I am satisfied that most of the reviewers’ concerns have been adequately addressed. However, one essential methodological point remains unresolved. Reviewer 1 requested clarification regarding the distributional functional form and the specific model fitted in your ARIMAX framework. In particular, the reviewer noted, “It is also not quite clear which distributional functional form and model was fitted. Poisson with ARIMA-based model for count outcomes, OLS ARIMA-based model, etc. These details must be provided.” This information is critical for ensuring transparency, reproducibility, and appropriate interpretation of your modelling approach. To proceed toward acceptance, please revise the manuscript to:

• Explicitly state the distributional assumptions underlying the model(s) used.

• Clarify whether the ARIMAX specification was implemented using a Poisson, quasi-Poisson, negative binomial, OLS, or other framework.

• Provide sufficient methodological detail for readers to understand and reproduce the modelling strategy, including any justification for the chosen distributional form.

Given that all other issues have been satisfactorily addressed, this clarification constitutes a minor revision. No additional reviewer input will be required once this point is resolved.

Dear editor:

We thank you and the reviewer for this important methodological concern about the distributional assumptions of the final fitted model. Thus, as requested, we have updated the 'Time series analysis' section (Lines 211–224) to explicitly state that a Gaussian ARIMAX framework was used; entry in lines 54-55 was also corrected.

We have clarified that the model assumes a Normal distribution for the error terms and that this assumption was validated via residual diagnostics, confirming that a discrete distributional form (e.g., Poisson) was not required for this dataset. We marked in blue the new entries.

Revised Manuscript Text (Abstract 54–55)

… with an AR(6) and seasonal MA(1) noise with expression: (1 − 𝜙₁ 𝐵 − 𝜙₂ 𝐵² − . . . − 𝜙₆ 𝐵⁶) (1ₜ − 𝐵ₜ₋₁₂) = 𝜀ₜ + 𝛩₁ 𝜀ₜ₋₁₂ with 𝜀 ~N(0, σ²).

Revised Manuscript Text (Lines 211–224)

The behavior of hepatitis B virus time series analysis from 2005–2020 was studied and used to identify and fit different plausible ARIMA models. Then, known data from 2021 was used for model testing and finally forecast was performed for 2022. The best fit was obtained with Model ARIMA (6, 0, 0) (0, 1, 1)_12, which incorporates information of six previous months and one seasonal innovation term (Figure 2). This model was implemented using a Gaussian distributional functional form, where the parameters were estimated via Maximum Likelihood Estimation (MLE). While the data consists of counts, the volume of monthly cases was sufficient to support a continuous approximation; the assumption that the residuals (𝜀ₜ) follow a Normal distribution with constant variance was validated through post-estimation diagnostic testing of the residuals. The functional form of this model captures the series autoregressive dynamics with an AR(6) and a seasonal noise with seasonal MA(1) with expression: (1 − 𝜙₁𝐵 − 𝜙₂𝐵² − . . . − 𝜙₆𝐵⁶)(𝐵ₜ − 𝐵ₜ₋₁₂) = 𝜀ₜ +𝛩₁ 𝜀ₜ₋₁₂ with 𝜀 ~N(0, σ²).

Attachment Submitted filename: reply_to_editor_reviwer.docx

# Peer review: Decision Letter 2

**DOI:** 10.1371/journal.pone.0329751.r005

**Authors:** Yordanis Enríquez Canto, Yordanis Enríquez Canto, Yordanis Enríquez Canto

3 Mar 2026

PONE-D-25-37298R2

Modelling Hepatitis B virus related hospital discharges in Spain: ARIMAX based liver disease forecasting tool for hospital workload and mortality progression

PLOS One

Dear Dr. Torner,

Thank you for submitting your manuscript to PLOS ONE. After careful consideration, we feel that it has merit but does not fully meet PLOS ONE’s publication criteria as it currently stands. Therefore, we invite you to submit a revised version of the manuscript that addresses the points raised during the review process.

==================================

The manuscript has improved substantially, and most issues raised in the first round have been satisfactorily addressed. However, one essential methodological concern remains unresolved and requires further clarification before the manuscript can proceed.

A key point raised by Reviewer 1 concerns the appropriateness of applying a Gaussian ARIMAX model to count-based outcome data. In your response, you indicate that the model was implemented under a Normal error assumption and that residual diagnostics supported this choice. While this information is helpful, it does not fully address the reviewer’s concern.

The issue raised is not limited to residual normality or homoscedasticity. It relates to fundamental distributional and structural properties of count data, which differ from those assumed by classical Gaussian ARIMA models.

=================================

Please submit your revised manuscript by Apr 17 2026 11:59PM. If you will need more time than this to complete your revisions, please reply to this message or contact the journal office at plosone@plos.org. When you're ready to submit your revision, log on to https://www.editorialmanager.com/pone/ and select the 'Submissions Needing Revision' folder to locate your manuscript file.

Please include the following items when submitting your revised manuscript:

- A letter that responds to each point raised by the academic editor and reviewer(s). You should upload this letter as a separate file labeled 'Response to Reviewers'.
- A marked-up copy of your manuscript that highlights changes made to the original version. You should upload this as a separate file labeled 'Revised Manuscript with Track Changes'.
- An unmarked version of your revised paper without tracked changes. You should upload this as a separate file labeled 'Manuscript'.

If you would like to make changes to your financial disclosure, please include your updated statement in your cover letter. Guidelines for resubmitting your figure files are available below the reviewer comments at the end of this letter.

If applicable, we recommend that you deposit your laboratory protocols in protocols.io to enhance the reproducibility of your results. Protocols.io assigns your protocol its own identifier (DOI) so that it can be cited independently in the future. For instructions see: https://journals.plos.org/plosone/s/submission-guidelines#loc-laboratory-protocols. Additionally, PLOS ONE offers an option for publishing peer-reviewed Lab Protocol articles, which describe protocols hosted on protocols.io. Read more information on sharing protocols at https://plos.org/protocols?utm_medium=editorial-email&utm_source=authorletters&utm_campaign=protocols.

We look forward to receiving your revised manuscript.

Kind regards,

Yordanis Enríquez Canto, Ph.D.

Academic Editor

PLOS One

Journal Requirements:

If the reviewer comments include a recommendation to cite specific previously published works, please review and evaluate these publications to determine whether they are relevant and should be cited. There is no requirement to cite these works unless the editor has indicated otherwise.

Additional Editor Comments:

Thank you for your continued work on the manuscript and for addressing the majority of the issues raised in the first review round. One key methodological point, however, remains unresolved and requires clarification before the manuscript can proceed.

The remaining concern relates to the suitability of applying a Gaussian ARIMAX model to monthly count data. While your response explains that residual diagnostics supported the Normal error assumption, this does not fully address the issue raised by Reviewer 1. The question is not limited to residual behavior but to the fundamental properties of count outcomes—non‑negativity, discreteness, and typically multiplicative dynamics—which are well documented in the statistical literature on time‑series modeling for counts.

To ensure methodological soundness and reproducibility, we ask that you either:

Re-estimate the analysis using an appropriate count-based time-series model (e.g., Poisson, Negative Binomial, quasi-Poisson, INGARCH, or GLM-based ARIMA), with justification; or

Provide a rigorous statistical justification for the use of a Gaussian ARIMAX model in this context, supported by formal evidence beyond residual normality (e.g., assessment of non-negativity of predictions, evaluation of the continuous approximation, comparison with a count-based alternative).

Once this point is addressed, no further reviewer input will be required. We appreciate your efforts to strengthen the manuscript and look forward to your revised submission.

[Note: HTML markup is below. Please do not edit.]

Reviewers' comments:

Reviewer's Responses to Questions

**Comments to the Author**

1. If the authors have adequately addressed your comments raised in a previous round of review and you feel that this manuscript is now acceptable for publication, you may indicate that here to bypass the “Comments to the Author” section, enter your conflict of interest statement in the “Confidential to Editor” section, and submit your "Accept" recommendation.

Reviewer #1: All comments have been addressed

**********

2. Is the manuscript technically sound, and do the data support the conclusions?

The manuscript must describe a technically sound piece of scientific research with data that supports the conclusions. Experiments must have been conducted rigorously, with appropriate controls, replication, and sample sizes. The conclusions must be drawn appropriately based on the data presented.

Reviewer #1: No

**********

3. Has the statistical analysis been performed appropriately and rigorously?

Reviewer #1: No

**********

4. Have the authors made all data underlying the findings in their manuscript fully available?

The PLOS Data policy requires authors to make all data underlying the findings described in their manuscript fully available without restriction, with rare exception (please refer to the Data Availability Statement in the manuscript PDF file). The data should be provided as part of the manuscript or its supporting information, or deposited to a public repository. For example, in addition to summary statistics, the data points behind means, medians and variance measures should be available. If there are restrictions on publicly sharing data—e.g. participant privacy or use of data from a third party—those must be specified.

Reviewer #1: Yes

**********

5. Is the manuscript presented in an intelligible fashion and written in standard English?

PLOS ONE does not copyedit accepted manuscripts, so the language in submitted articles must be clear, correct, and unambiguous. Any typographical or grammatical errors should be corrected at revision, so please note any specific errors here.

Reviewer #1: Yes

**********

6. Review Comments to the Author

Please use the space provided to explain your answers to the questions above. You may also include additional comments for the author, including concerns about dual publication, research ethics, or publication ethics. (Please upload your review as an attachment if it exceeds 20,000 characters)

Reviewer #1: Authors responds

“We have clarified that the model assumes a Normal distribution for the error terms and that this assumption was validated via residual diagnostics, confirming that a discrete distributional form (e.g., Poisson) was not required for this dataset. We marked in blue the new entries.

Revised Manuscript Text (Abstract 54–55)

… with an AR(6) and seasonal MA(1) noise with expression: (1 − ₁ − ₂ ² − . . . − ₆ ⁶) ( ₜ − ₜ₋₁₂) = ₜ + ₁ ₜ₋₁₂ with ~N(0, σ²).

Revised Manuscript Text (Lines 211–224)

The behavior of hepatitis B virus time series analysis from 2005–2020 was studied and used to identify and fit different plausible ARIMA models. Then, known data from 2021 was used for model testing and finally forecast was performed for 2022. The best fit was obtained with Model ARIMA (6, 0, 0) (0, 1, 1)_12, which incorporates information of six previous months and one seasonal innovation term (Figure 2). This model was implemented using a Gaussian distributional functional form, where the parameters were estimated via Maximum Likelihood Estimation (MLE). While the data consists of counts, the volume of monthly cases was sufficient to support a continuous approximation; the assumption that the residuals ( ₜ) follow a Normal distribution with constant variance was validated through post-estimation diagnostic testing of the residuals. The functional form of this model captures the series autoregressive dynamics with an AR(6) and a seasonal noise with seasonal MA(1) with expression: (1 − ₁ − ₂ ² − . . . − ₆ ⁶)( ₜ − ₜ₋₁₂) = ₜ + ₁ ₜ₋₁₂ with ~N(0, σ²).”

I humbly disagree with the authors' position: First of all, OLS requires that the residuals be normally distributed, not the count model's functional form. Authors admitted in their first statement that the normal distribution for the error terms assumption was validated via residual diagnostics, and that is all the more reason why OLS can be used in this situation at all. Linear regression (OLS) is generally inappropriate for modeling count outcomes (e.g., hospital discharges, number of accidents, hospital visits, defects, crimes) for several fundamental statistical reasons. Count data have structural properties that violate key OLS assumptions. Assume that the first two assumptions were validated (No violation of the vormality Assumption and Heteroskedasticity (Non-Constant Variance) as stated by the authors:

What about the following key issues of modelling count data with linear regtression models which are so critical:

High Possibility of Negative Predictions: OLS produces predictions over the entire real line: (-∞,+∞). But count outcome models must satisfy: Y≥0. OLS can (and often does) produce negative predicted counts (negative hospital discharges) and non-integer predictions, which are conceptually invalid for count data.

Incorrect Functional Form (Additive Instead of Multiplicative). Linear regression assumes a linear relationship, while count processes often exhibit multiplicative relationships. The log-link structure ensures non-negativity, models proportional changes and reflects exponential growth patterns common in count processes. The OLS imposes an inappropriate additive structure.

Inefficiency and Inconsistency Under Misspecification: When the true conditional distribution is Poisson or Negative Binomial, OLS is not maximum likelihood and it is statistically inefficient

Poor Handling of Zero Inflation: Many count datasets exhibit excess zeros relative to Poisson/Negative binomial expectation. OLS cannot model zero inflation and capture structural zeros. That is all the more reason to use specialized models (Zero-Inflated Poisson, Hurdle models) designed for this structure where applicable.

Discreteness Ignored: Counts are discrete, but OLS treats the dependent variable as continuous. This leads to misrepresentation of probability mass, inappropriate inference procedures, and loss of distributional information

Finally, for count outcome measures, it is appropriate to use count outcomes, use Poisson Regression, Negative Binomial Regression (for overdispersion), quasi-Poisson, Zero-Inflated Models, and Hurdle Models where appropriate. All are members of the Generalized Linear Model (GLM) family. Kindly re-look at the model generating the results as it is a bit problematic to model a classical count outcome with linear models. Please use the appropriate models so inference from the study will be valid

**********

7. PLOS authors have the option to publish the peer review history of their article (what does this mean?). If published, this will include your full peer review and any attached files.

If you choose “no”, your identity will remain anonymous but your review may still be made public.

**Do you want your identity to be public for this peer review?**  For information about this choice, including consent withdrawal, please see our Privacy Policy.

Reviewer #1: No

**********

[NOTE: If reviewer comments were submitted as an attachment file, they will be attached to this email and accessible via the submission site. Please log into your account, locate the manuscript record, and check for the action link "View Attachments". If this link does not appear, there are no attachment files.]

To ensure your figures meet our technical requirements, please review our figure guidelines: https://journals.plos.org/plosone/s/figures

You may also use PLOS’s free figure tool, NAAS, to help you prepare publication quality figures: https://journals.plos.org/plosone/s/figures#loc-tools-for-figure-preparation.

NAAS will assess whether your figures meet our technical requirements by comparing each figure against our figure specifications.

# Author comment: Author response to Decision Letter 3

**DOI:** 10.1371/journal.pone.0329751.r006

9 Apr 2026

Dear Editor and Reviewer 1,

We sincerely thank you for the opportunity to further clarify our methodological approach. We appreciate Reviewer 1’s detailed and insightful comments regarding the structural properties of count data, including non-negativity, discreteness, and multiplicative dynamics. We fully agree that a naive application of Gaussian ARIMA to raw, low level counts is inappropriate.

However, we wish to clarify that our modelling approach was not conducted on raw counts, but within a Variance-Stabilizing Transformation (VST) framework specifically designed to map discrete count processes into a Gaussian-compliant space. While this step was implemented in the original analysis, we acknowledge it was not explicitly documented; we have now corrected this in the revised manuscript.

We provide the following rigorous statistical justification for the use of the approximate Gaussian ARIMAX methodology:

Variance-stabilizing transformation (VST): To address the issues of heteroscedasticity and the multiplicative dynamics inherent in count data, we applied a square-root transformation (Box–Cox with λ=0.5) prior to estimation. As established by Bartlett (1936), the square-root transformation is the optimal VST for Poisson-distributed data, as it transforms the mean-variance relationship (where Var(Y)=μ) into an approximately constant variance (Var(√Y) ≈0.25). Lines 167-172

This transformation specifically addresses the Reviewer’s concerns by: linearizing multiplicative dynamics, because it mitigates the mismatch between the additive structure of ARIMAX and the multiplicative nature of counts, and by normalizing the error structure: It allows the use of Gaussian Likelihood while maintaining consistency with the underlying count process (Shumway and Stoffer, 2016).

Suitability of the continuous approximation for count data: While hospital discharges are discrete, our aggregated monthly counts range from 10 to 55. For a Poisson process P(λ), the distribution approaches normality as λ increases. It is a well-accepted convention in time series analysis that when λ≥10, the loss of information incurred by a continuous approximation is negligible (Box et al., 2005; Box et al., 2008). In our dataset, the mean is sufficiently far from the zero boundary to treat the transformed series as a continuous Gaussian process without risking "boundary effects." Lines 225-233

Consistency via quasi-maximum likelihood estimation (QMLE): Parameter estimation was conducted using a Gaussian likelihood function. Under standard regularity conditions, this approach yields consistent parameter estimates for ARMA-type models even when the true conditional distribution deviates from Gaussianity, provided the conditional mean and variance are correctly specified and the innovations have finite variance (Hamilton, 1994; Tsay, 2010). Lines 335-340

Residual diagnostics: As noted in our previous review, post-estimation graphical tools and diagnostic testing, including assessments of autocorrelation (Box–Ljung test), homoscedasticity, and normality, indicated that the residuals behave as Gaussian white noise. These results support the adequacy of the approximate Gaussian ARIMAX structure in successfully capturing the temporal dynamics of the series. Lines 235-237

Non-Negativity of predictions: A primary concern raised was the possibility of negative predictions. We have verified that: all in sample fits, out-of-sample predictions and forecasts were strictly non-negative. Given the mean (μ) and the estimated variance (σ^2) of our transformed series, the probability of the model generating a negative value is statistically negligible (the zero boundary lies far away from the mean). This indicates that the model respected the natural bounds of the data despite not imposing an explicit non-negativity constraint. Lines 280-287

Predictive performance: The approximate ARIMAX model achieved good predictive accuracy (MAPE = 13.9%), providing empirical evidence that the Gaussian approximation, when combined with a VST, yields a robust representation of the system's underlying data-generating process. Lines 280-287

Conclusion:

In light of these points, we believe the approximate Gaussian √Y-ARIMAX approach is not only appropriate but provides a parsimonious and rigorously justified alternative to specialized count models (which can often suffer from convergence issues or over-parameterization in small-to-moderate sample sizes). Thus, as our results confirm that the transformation effectively addressed the discreteness and heteroscedasticity of the series, this approach stands as a statistically sound and parsimonious methodology for the research objective.

Specific Manuscript Edits under this revision

Note: We have updated (red font) the Material and Methods and the Discussion to explicitly detail the statistical rationale for the square root transformation and the adequacy of the Gaussian approximation for this study. Additional clarifying review inputs are clearly reflected in red font.

Author Contributions:

Abstract: (methods y results)

Material and methods section: (statistical methodology)

Results: (, time series anal:ysis,)

Discussion

Mention the Transformation: "To account for the discrete, non-negative nature of hospital discharge counts and to stabilize the variance, a square-root transformation was applied to the dependent variable ($y_t' = \sqrt{y_t}$) prior to ARIMAX modeling (Bartlett, 1936)."

Mention the Boundary: "Given that monthly counts exceeded 10 in all periods, a Gaussian approximation was deemed appropriate (Box et al., 2015)."

Refine the Residuals Section: "Residual diagnostics confirmed the adequacy of this transformation, showing no evidence of heteroscedasticity or non-normality."

References added or updated

Bartlett, M.S. (1936). The Square Root Transformation in Analysis of Variance. Supplement to the Journal of the Royal Statistical Society, 3(1), 68–78. https://doi-org.recursos.biblioteca.upc.edu/10.2307/2983678

Box GEP, Hunter JS, Hunter WG (2005) Statistics for experimenters. 2nd ed. Hoboken (NJ): John Wiley & Sons.

Box GEP, Jenkins GM, Reinsel GC (2008) Time series analysis: forecasting and control. 4th ed. Hoboken (NJ): John Wiley & Sons.

Shumway RH, Stoffer DS (2016) Time series analysis and its applications: with R examples. Available from: http://www.stat.ucla.edu/~frederic/415/S23/tsa4.pdf

Hamilton JD (1994) Time series analysis. Princeton (NJ): Princeton University Press.

Tsay RS (2010) Analysis of financial time series. 3rd ed. Hoboken (NJ): John Wiley & Sons.

Attachment Submitted filename: Response_to_reviewers_auresp_3.docx

# Peer review: Decision Letter 3

**DOI:** 10.1371/journal.pone.0329751.r007

**Authors:** Yordanis Enríquez Canto, Yordanis Enríquez Canto, Yordanis Enríquez Canto, Yordanis Enríquez Canto

18 May 2026

Modelling Hepatitis B virus related hospital discharges in Spain: ARIMAX based liver disease forecasting tool for hospital workload and mortality progression

PONE-D-25-37298R3

Dear Dr. Torner,

We’re pleased to inform you that your manuscript has been judged scientifically suitable for publication and will be formally accepted for publication once it meets all outstanding technical requirements.

Within one week, you’ll receive an e-mail detailing the required amendments. When these have been addressed, you’ll receive a formal acceptance letter and your manuscript will be scheduled for publication.

An invoice will be generated when your article is formally accepted. Please note, if your institution has a publishing partnership with PLOS and your article meets the relevant criteria, all or part of your publication costs will be covered. Please make sure your user information is up-to-date by logging into Editorial Manager at Editorial Manager® and clicking the ‘Update My Information' link at the top of the page. For questions related to billing, please contact billing support.

If your institution or institutions have a press office, please notify them about your upcoming paper to help maximize its impact. If they’ll be preparing press materials, please inform our press team as soon as possible -- no later than 48 hours after receiving the formal acceptance. Your manuscript will remain under strict press embargo until 2 pm Eastern Time on the date of publication. For more information, please contact onepress@plos.org.

Kind regards,

Yordanis Enríquez Canto, Ph.D.

Academic Editor

PLOS One

Additional Editor Comments (optional):

Reviewers' comments:

Reviewer's Responses to Questions

**Comments to the Author**

1. If the authors have adequately addressed your comments raised in a previous round of review and you feel that this manuscript is now acceptable for publication, you may indicate that here to bypass the “Comments to the Author” section, enter your conflict of interest statement in the “Confidential to Editor” section, and submit your "Accept" recommendation.

Reviewer #2: All comments have been addressed

**********

2. Is the manuscript technically sound, and do the data support the conclusions?

The manuscript must describe a technically sound piece of scientific research with data that supports the conclusions. Experiments must have been conducted rigorously, with appropriate controls, replication, and sample sizes. The conclusions must be drawn appropriately based on the data presented.

Reviewer #2: Yes

**********

3. Has the statistical analysis been performed appropriately and rigorously?

Reviewer #2: Yes

**********

4. Have the authors made all data underlying the findings in their manuscript fully available?

The PLOS Data policy requires authors to make all data underlying the findings described in their manuscript fully available without restriction, with rare exception (please refer to the Data Availability Statement in the manuscript PDF file). The data should be provided as part of the manuscript or its supporting information, or deposited to a public repository. For example, in addition to summary statistics, the data points behind means, medians and variance measures should be available. If there are restrictions on publicly sharing data—e.g. participant privacy or use of data from a third party—those must be specified.

Reviewer #2: Yes

**********

5. Is the manuscript presented in an intelligible fashion and written in standard English?

PLOS ONE does not copyedit accepted manuscripts, so the language in submitted articles must be clear, correct, and unambiguous. Any typographical or grammatical errors should be corrected at revision, so please note any specific errors here.

Reviewer #2: Yes

**********

6. Review Comments to the Author

Please use the space provided to explain your answers to the questions above. You may also include additional comments for the author, including concerns about dual publication, research ethics, or publication ethics. (Please upload your review as an attachment if it exceeds 20,000 characters)

Reviewer #2: The authors have addressed and incorporated the recommendations provided by the reviewer. The revised version reflects the suggested changes, and the modifications made have improved the clarity and overall quality of the manuscript. We appreciate the authors’ efforts in carefully considering the comments and revising the manuscript accordingly.

**********

7. PLOS authors have the option to publish the peer review history of their article (what does this mean?). If published, this will include your full peer review and any attached files.

If you choose “no”, your identity will remain anonymous but your review may still be made public.

**Do you want your identity to be public for this peer review?**  For information about this choice, including consent withdrawal, please see our Privacy Policy.

Reviewer #2: No

**********

# Editor report: Acceptance letter

**DOI:** 10.1371/journal.pone.0329751.r008

**Authors:** Yordanis Enríquez Canto, Yordanis Enríquez Canto, Yordanis Enríquez Canto, Yordanis Enríquez Canto

PONE-D-25-37298R3

PLOS One

Dear Dr. Torner,

I'm pleased to inform you that your manuscript has been deemed suitable for publication in PLOS One. Congratulations! Your manuscript is now being handed over to our production team.

At this stage, our production department will prepare your paper for publication. This includes ensuring the following:

* All references, tables, and figures are properly cited

* All relevant supporting information is included in the manuscript submission,

* There are no issues that prevent the paper from being properly typeset

You will receive further instructions from the production team, including instructions on how to review your proof when it is ready. Please keep in mind that we are working through a large volume of accepted articles, so please give us a few days to review your paper and let you know the next and final steps.

Lastly, if your institution or institutions have a press office, please let them know about your upcoming paper now to help maximize its impact. If they'll be preparing press materials, please inform our press team within the next 48 hours. Your manuscript will remain under strict press embargo until 2 pm Eastern Time on the date of publication. For more information, please contact onepress@plos.org.

You will receive an invoice from PLOS for your publication fee after your manuscript has reached the completed accept phase. If you receive an email requesting payment before acceptance or for any other service, this may be a phishing scheme. Learn how to identify phishing emails and protect your accounts at https://explore.plos.org/phishing.

If we can help with anything else, please email us at customercare@plos.org.

Thank you for submitting your work to PLOS ONE and supporting open access.

Kind regards,

PLOS ONE Editorial Office Staff

on behalf of

Prof. Yordanis Enríquez Canto

Academic Editor

PLOS One
